# Heterochronic parabiosis reprograms the mouse brain transcriptome by shifting aging signatures in multiple cell types

**DOI:** 10.1038/s43587-023-00373-6

**Published:** 2023-03-09

**Authors:** Methodios Ximerakis, Kristina M. Holton, Richard M. Giadone, Ceren Ozek, Monika Saxena, Samara Santiago, Xian Adiconis, Danielle Dionne, Lan Nguyen, Kavya M. Shah, Jill M. Goldstein, Caterina Gasperini, Ioannis A. Gampierakis, Scott L. Lipnick, Sean K. Simmons, Sean M. Buchanan, Amy J. Wagers, Aviv Regev, Joshua Z. Levin, Lee L. Rubin

**Affiliations:** 1 Department of Stem Cell and Regenerative Biology, Harvard University, Cambridge, MA USA; 2 Harvard Stem Cell Institute, Cambridge, MA USA; 3 Stanley Center for Psychiatric Research, Broad Institute of MIT and Harvard, Cambridge, MA USA; 4 Klarman Cell Observatory, Broad Institute of MIT and Harvard, Cambridge, MA USA; 5 Joslin Diabetes Center, Boston, MA USA; 6 Paul F. Glenn Center for the Biology of Aging, Harvard Medical School, Boston, MA USA; 7 Howard Hughes Medical Institute, Koch Institute of Integrative Cancer Research, Department of Biology, Massachusetts Institute of Technology, Cambridge, MA USA

**Keywords:** Neural ageing, Neuro-vascular interactions, Blood-brain barrier, Alzheimer's disease, Gene regulatory networks

## Abstract

Aging is a complex process involving transcriptomic changes associated with deterioration across multiple tissues and organs, including the brain. Recent studies using heterochronic parabiosis have shown that various aspects of aging-associated decline are modifiable or even reversible. To better understand how this occurs, we performed single-cell transcriptomic profiling of young and old mouse brains after parabiosis. For each cell type, we cataloged alterations in gene expression, molecular pathways, transcriptional networks, ligand–receptor interactions and senescence status. Our analyses identified gene signatures, demonstrating that heterochronic parabiosis regulates several hallmarks of aging in a cell-type-specific manner. Brain endothelial cells were found to be especially malleable to this intervention, exhibiting dynamic transcriptional changes that affect vascular structure and function. These findings suggest new strategies for slowing deterioration and driving regeneration in the aging brain through approaches that do not rely on disease-specific mechanisms or actions of individual circulating factors.

## Abstract (web summary)

Single-cell transcriptomic profiling of young and old mouse brains following heterochronic parabiosis shows regulation of several canonical hallmarks of aging by a shift in age-induced changes of the transcriptome in a cell-type-specific manner.

## Main

Aging is a complicated process that is far from being completely understood, although a number of hallmarks have been recognized^1^. The brain itself is affected substantially by aging, with processes such as cellular respiration, protein synthesis, oxidative stress, neurotransmission, myelination, neurogenesis, inflammation and blood flow being compromised^2,3^. None the less, a series of recent observations demonstrate that several aspects of aging can be delayed or even reversed by a variety of interventions, including exercise^4,5^, caloric restriction^6^, elimination of senescent cells^7,8^, administration of rapamycin^9^ or metformin^10^, transient cell reprogramming^11^ and young bone marrow transplantation^12^.

One of the most robust methods of improving the function of aging tissues is that of heterochronic parabiosis, a surgical procedure whereby young and old mice are joined together so that they share a common circulatory system^13,14^. Multiple publications have led to the surprising conclusion that exposure of old mice to the young circulatory environment improves the function of various tissues and organs^15–21^, including the central nervous system (CNS)^22–25^. In the CNS specifically, studies from our lab^25,26^ and others^24,27–30^ have shown that circulating factors in young blood stimulate functional improvement in aged and diseased brains. Conversely, it has been shown that systemic factors in old blood drive aging phenotypes in young tissues^22,31,32^, including the brain. The molecular underpinnings of these changes remain to be fully elucidated, but a great deal of recent work has focused on measuring age-related changes in serum blood proteins^33^, primarily based on the hypothesis that function-improving factors decline with age. Although this is a plausible approach^25,28,29,34–36^, it ignores nonprotein factors (blood cells^23^, exosomes, lipids) and factors that either do or do not change in unexpected directions with aging.

Following our previously published work employing single-cell RNA-sequencing (scRNA-seq), which describes changes that occur in the brain during aging^37^, we have now quantified changes in the transcriptomes of young and old mouse brains after parabiosis. The comprehensive single-cell datasets we generated allowed us to detect brain cell types, the transcriptional states of which are affected by parabiosis. We also identified changes in inter-/intracellular molecular pathways, gene regulatory networks, cell–cell interactions and senescence. Endothelial cells (ECs) were found to be highly affected by parabiosis, exhibiting strong dynamic changes in their transcriptome, with several of these validated by orthogonal assays. Overall, this work shows that heterochronic parabiosis regulates several canonical hallmarks of aging^1,3^ by shifting aging-induced changes of the transcriptome in a cell-type-specific manner.

## Results

### Single-cell profiling to study rejuvenation and aging acceleration

**Fig. 1:**
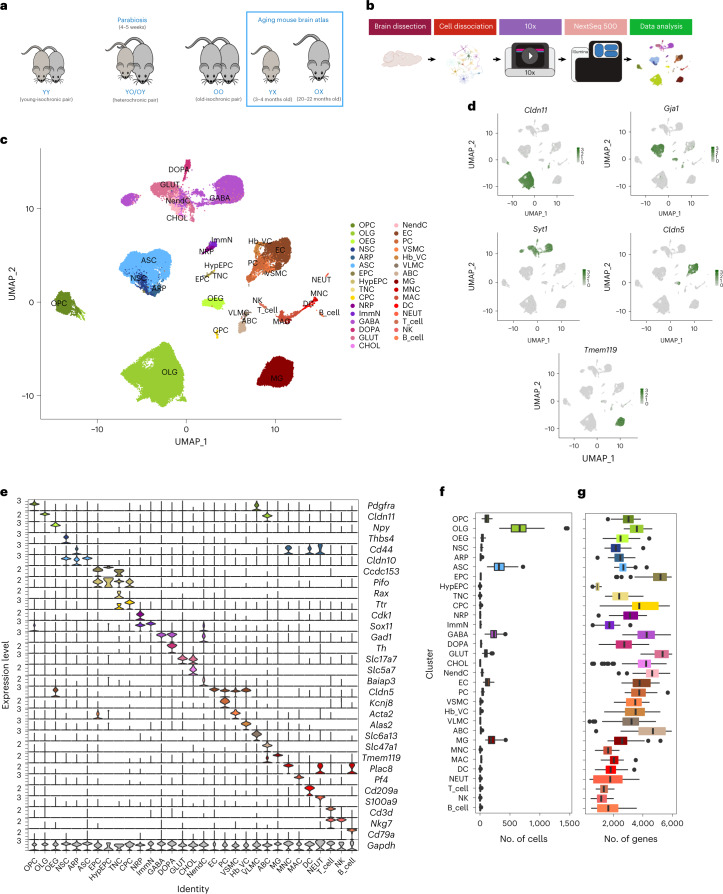
Overview of the single-cell sequencing analysis. **a**, Schematic representation of the animal types used in the present study. Sequencing data from isochronic (YY and OO) and heterochronic (YO and OY) parabiosis pairs were generated and integrated with sequencing data from young (YX) and old (OX) unpaired mice from our previous work^37^ which were generated simultaneously with those of the parabionts. **b**, Schematic representation of the experimental workflow (see Methods for details). **c**, UMAP projection of 105,329 cells across 31 clusters derived from 34 parabionts (7 YY, 9 YO, 7 OO and 11 OY) and 16 unpaired animals (8 YX and 8 OX). For the cell-type abbreviations please see the text and Methods (Supplementary Table 1). **d**, UMAP projection of five major cell populations showing the expression of representative, well-known, cell-type-specific marker genes (OLGs: *Cldn11*; ASCs: *Gja1*; NEUR: *Syt1*; ECs: *Cldn5*; MGs: *Tmem119*). The numbers reflect the number of nCount RNA (UMI) detected for the specified gene for each cell. **e**, Violin plot showing the distribution of expression levels of well-known, representative, cell-type-enriched, marker genes across all 31 distinct cell types. **f**,**g**, Boxplot showing the distribution over *n* = 50 biologically independent animals of the number of detected cells per cell type (**f**) or number of detected genes per cell type (**g**). Boxplot minimum is the smallest value within 1.5× the interquartile range (IQR) below the 25th percentile and maximum is the largest value within 1.5× the IQR above the 75th percentile. Boxplot center is the 50th percentile (median) and box bounds are the 25th and 75th percentiles. Outliers are >1.5× and <3× the IQR. Panel **b** was created with BioRender.com.

**Extended Data Fig. 1:**
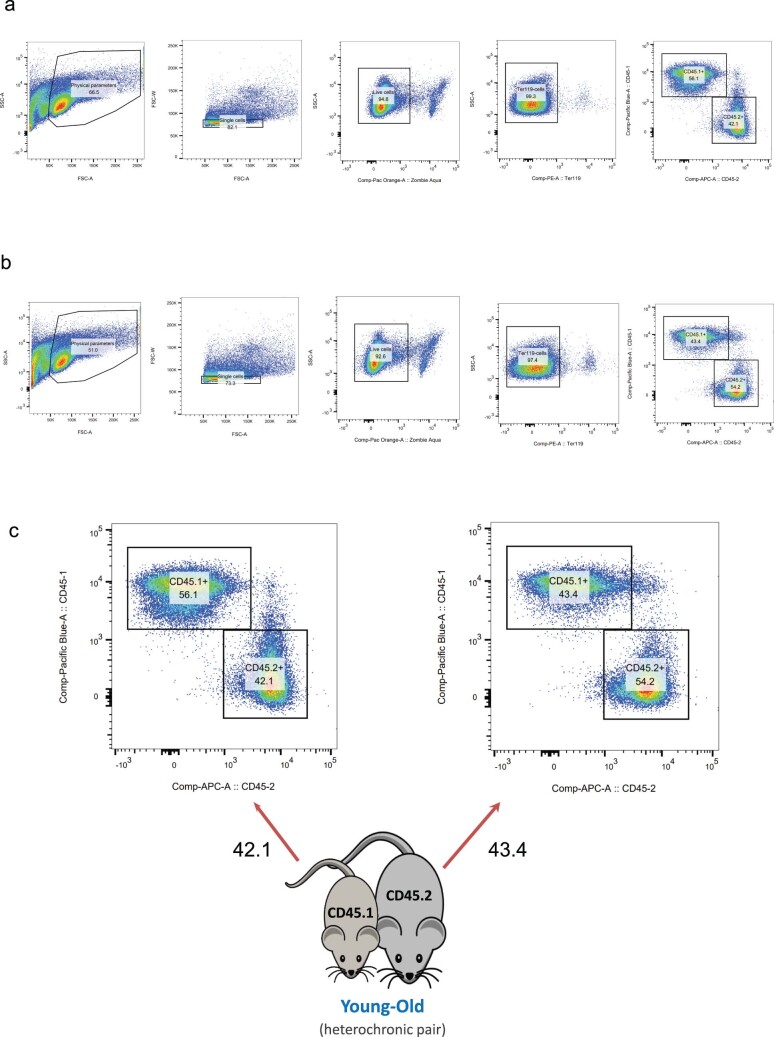
Confirmation of blood chimerism. Representative flow cytometry analysis of CD45.1 and CD45.2 expression markers on splenocytes isolated from (**a**) young (CD45.1^+^) and (**b**) old mice (CD45.2^+^) following heterochronic parabiosis. **c)** The percentage of donor-derived blood cells from one partner in the spleen of the other partner is depicted by arrows.

**Extended Data Fig. 2:**
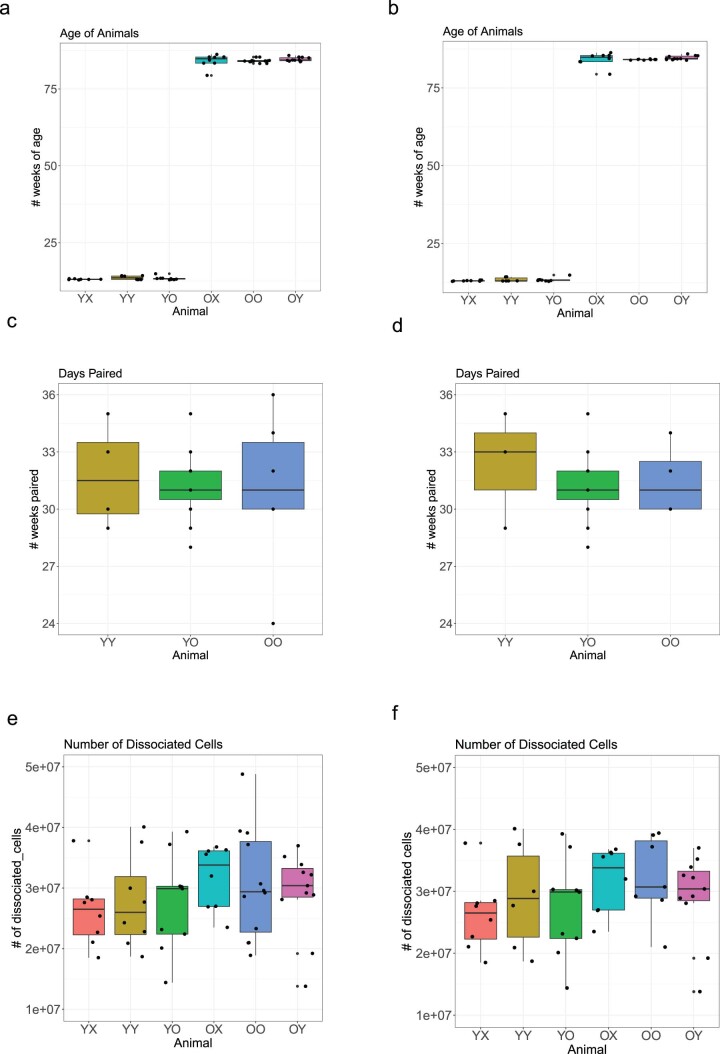
Sample metrics. Profiling of animals and their derived brain cells used for sequencing, before (N = 56) (a,c,e) and after (n = 50) (b,d,f) quality control filtering in which certain animals were omitted (see Methods). Boxplot minimum is the smallest value within 1.5 times the interquartile range below the 25^th^ percentile, maximum is the largest value within 1.5 times the interquartile range above the 75^th^ percentile. Boxplot center is the 50^th^ percentile (median), box bounds are the 25^th^ and 75^th^ percentile. Outliers are >1.5 times and < 3 times the interquartile range. **a-b**. Age of mice in weeks prior to parabiosis surgeries. **c-d**. Number of days joined across parabiotic pairs. **e-f**. Number of dissociated cells analyzed per brain across all animal types.

**Extended Data Fig. 3:**
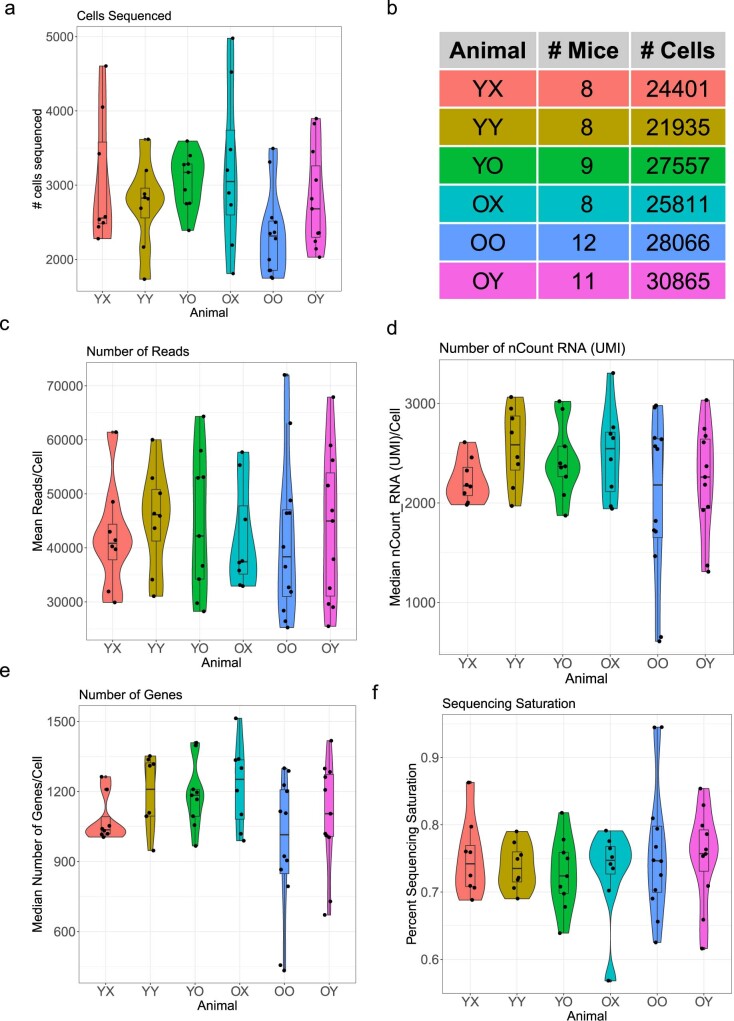
Sequencing metrics. Violin plots with boxplots showing sequencing metrics of the distribution of animals from all sequenced animal types. Each dot represents one animal. Boxplot minimum is the smallest value within 1.5 times the interquartile range below the 25^th^ percentile, maximum is the largest value within 1.5 times the interquartile range above the 75^th^ percentile. Boxplot center is the 50^th^ percentile (median), box bounds are the 25^th^ and 75^th^ percentile. Outliers are >1.5 times and < 3 times the interquartile range. **a**. Number of cells sequenced by animal. **b**. Table of total number of animals and cells analyzed. **c**. Mean number of mapped reads per cell by animal. **d**. Median number of nCount RNA (UMI) detected per cell by animal. **e**. Median number of genes detected per cell by animal. **f**. Percent of sequencing saturation by animal.

**Extended Data Fig. 4:**
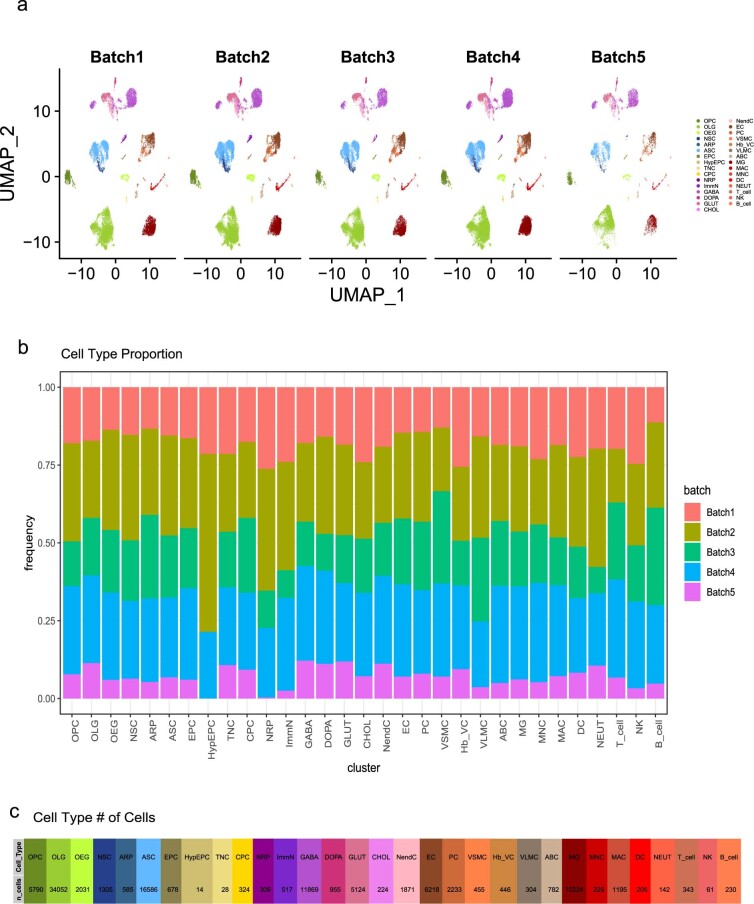
Distribution of 50 animals across 5 sequencing batches, with respect to cell clusters, and cell count. **a**. UMAP projection of color-coded batches over clusters that passed filtering criteria. **b**. Frequency of each color-coded batch representation in each cell type. All cell types are represented by cells from all batches, except for HypEPC in batch 5, probably due to its small size. **c**. Number of detected cells in each cell type.

**Extended Data Fig. 5:**
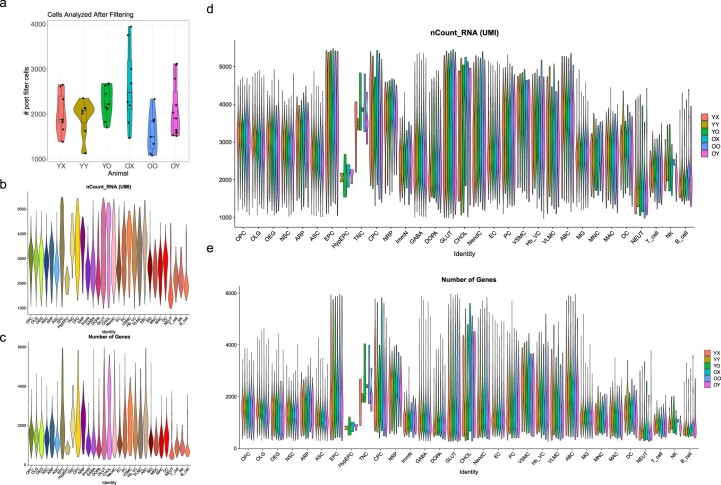
Primary data analysis. a. Violin plot and boxplot showing the number of cells analyzed by animal after cell filtering, in which all cells were successfully assigned to a specific cell type. Each dot represents one animal. Boxplot minimum is the smallest value within 1.5 times the interquartile range below the 25^th^ percentile, maximum is the largest value within 1.5 times the interquartile range above the 75^th^ percentile. Boxplot center is the 50^th^ percentile (median), box bounds are the 25^th^ and 75^th^ percentile. **b-e**. Violin plots showing QC metrics, plots in (b, c) showing aggregated data of cells of all brain types, while plots in (d, e) showing individual cell data separated by animal type: (b, d) showing nCount RNA (UMI) per cell type. (c, e) showing nFeature RNA (number of unique genes) detected per cell.

We employed high-throughput scRNA-seq to examine the transcriptional profiles of young and old mouse brains after parabiosis (Fig. 1a,b). We generated heterochronic pairs in which 3- to 4-month-old mice were joined with 20- to 22-month-old mice. We also generated age-matched isochronic pairs of young and old mice as controls. All pairs were maintained for 4–5 weeks before tissue collection and analysis. We confirmed successful parabiosis and establishment of blood crosscirculation as previously described^38^ (Extended Data Fig. 1). We dissociated the brain tissues using our recently developed protocol^37^ and analyzed the transcriptomes of 158,767 single cells (Extended Data Figs. 2 and 3). On stringent filtering and batch effect examination (Methods), we retained 105,329 cells, of which 67,992 cells derived from 34 parabionts (7 isochronic young (YY), 9 heterochronic young (YO), 7 isochronic old (OO), 11 heterochronic old (OY)) and 37,337 cells derived from 16 unpaired animals (8 young (YX) and 8 old (OX)) (Fig. 1 and Extended Data Figs. 4 and 5).

### Single-cell atlas reveals that cell types are preserved by parabiosis

By combining cells in unpaired and parabiotic brains and using cell markers from our previous work^37^, we identified 31 major cell types with distinct expression profiles (Fig. 1c). For a complete list of cell types and abbreviations, see Methods and Supplementary Table 1.

**Extended Data Fig. 6:**
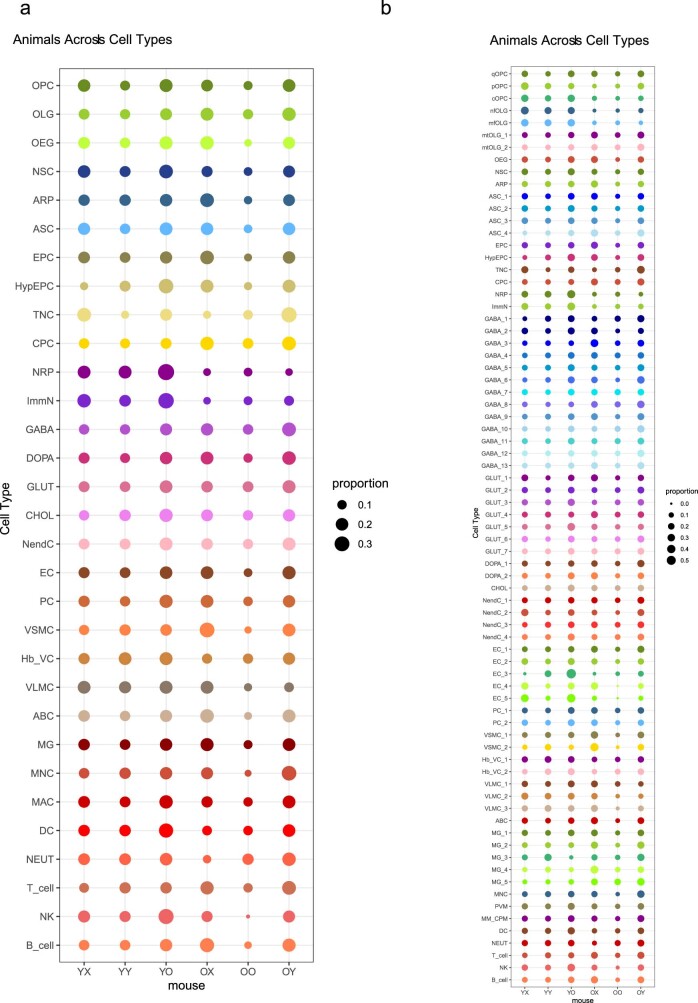
Representation of each animal type’s distribution within each cell type. **a**. Dot plot representation of each cell type’s representation by each animal type. Size of the dot is proportional to the number of cells contributed by each animal type within a cell type. **b**. Dot plot representation of each subpopulation’s representation by each animal type. Size of the dot is proportional to the number of cells contributed by each animal type within a subpopulation.

**Extended Data Fig. 7:**
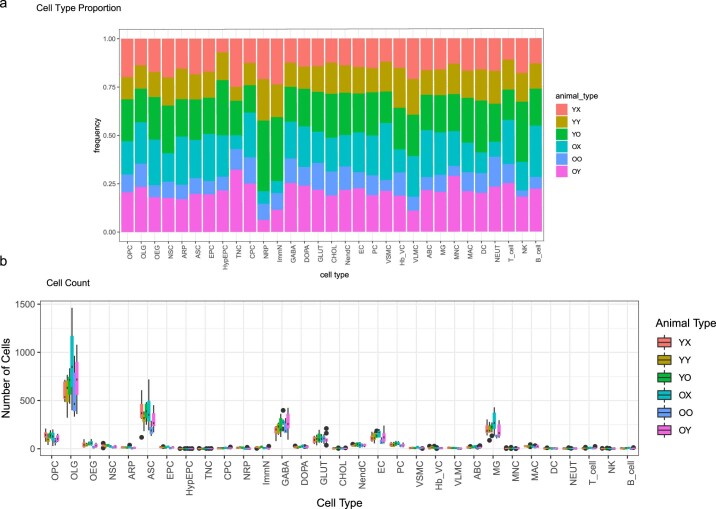
Cell type composition and cell count from each animal type. **a**. Frequency bar plot demonstrating composition of each cell type with respect to animal type. **b**. Boxplot of raw cell counts with respect to each animal. All animals contribute to all cell types. ANOVA p-values (one-tailed) for pairwise iterations can be found in Supplementary Table 2. The only comparisons with unadjusted p-values < 0.05 are: OOvOX: DC (p = 0.041), YOvYX DOPA (p = 0.022), YYvYX OEG (p = 0.046), ImmN (p = 0.045), and PC (p = 0.016), Boxplot minimum is the smallest value within 1.5 times the interquartile range below the 25th percentile, maximum is the largest value within 1.5 times the interquartile range above the 75th percentile. Boxplot center is the 50th percentile (median), box bounds are the 25th and 75th percentile. Outliers are >1.5 times and < 3 times the interquartile range.

We then examined the major markers for each cell type to ensure robustness of their transcriptional signatures. Each cell type expressed markers that match its cellular identity, as previously characterized^37^ (Fig. 1d,e). Unsupervised clustering also showed that the identified cell populations were represented in all batches and animal types (Extended Data Figs. 4a,b and 6), indicating that cell identity is preserved in parabiotic mice. We found that the proportions of each cell type represented in each group were only slightly smaller for the isochronic brains, which may be due to the smaller number of contributing animals (Extended Data Fig. 7). We quantified the difference in the total number of cells for each cell population by comparing the animal types using analysis of variance (ANOVA) with a threshold of *P* ≤ 0.05, confirming that parabiosis does not significantly alter the number of cells per cell population between animal types, except for dopaminergic neurons (DOPA) in young heterochronic versus young unpaired mice (*P* = 0.002) (Fig. 1f, Extended Data Figs. 4c and 7 and Supplementary Table 2). However, data analysis of cell proportion changes should be cautiously considered in single-cell sequencing studies, especially when tissue dissociation is used. Across the different types of cells, we observed the largest number of total detected genes in ependymocytes (EPCs), choroid plexus epithelial cells (CPCs), GABAergic neurons (GABA), glutamatergic neurons (GLUT), neuroendocrine cells (NendCs)^37^ and arachnoid barrier cells (ABCs) (Fig. 1g).

**Fig. 2:**
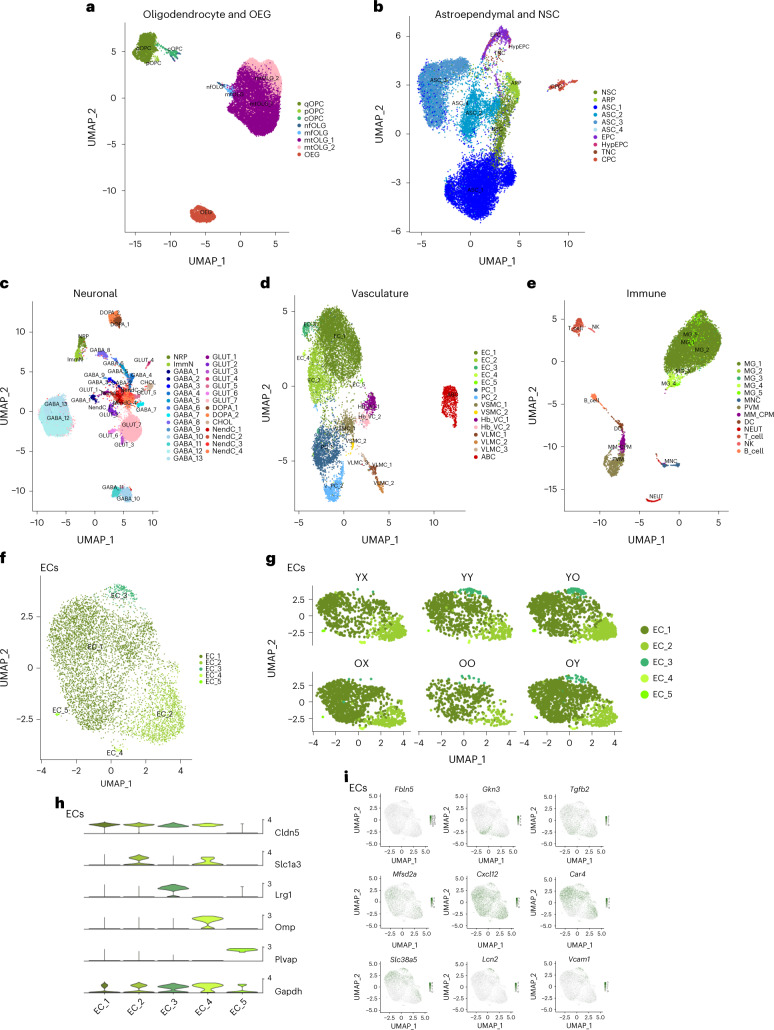
Characterization of cell types and subpopulations. **a**–**e**, Subpopulation analysis of cell types grouped in five distinct cell classes: OLG lineage and OEGs (*n* = 41,873 cells) (**a**), astroependymal cells and NSCs (*n* = 19,520 cells) (**b**), neuronal lineage (*n* = 20,869 cells) (**c**), vasculature cells (*n* = 10,438 cells) (**d**) and immune cells (*n* = 12,629 cells) (**e**). *q*, quiescent; *p*, proliferating; *c*, committed, *nf*, newly formed; *mf*, myelin-formin; *mt*, mature. **f**, UMAP subpopulation analysis of EC clusters (*n* = 6,218 cells). **g**, UMAP subpopulation of EC clusters, stratified by animal type. **h**, Violin plot of delineating markers of ECs, as *Cldn5*, *Slc1a3*, *Lrg1*, *Omp* and *Plvap*. **i**, UMAP overlay of EC zonation markers along the arteriovenous axis curated from the literature^41–43^. Markers in left-to-right order: large arteries: *Fbln5*; arterial: *Gkn3;* capillary–arterial: *Tgfb2*; capillary: *Mfsd2a*; capillary: *Cxcl12*; capillary–venous: *Car4*; venous: *Slc38a6*; large veins: *Lcn2*; and large vessels: *Vcam1*.

**Extended Data Fig. 8:**
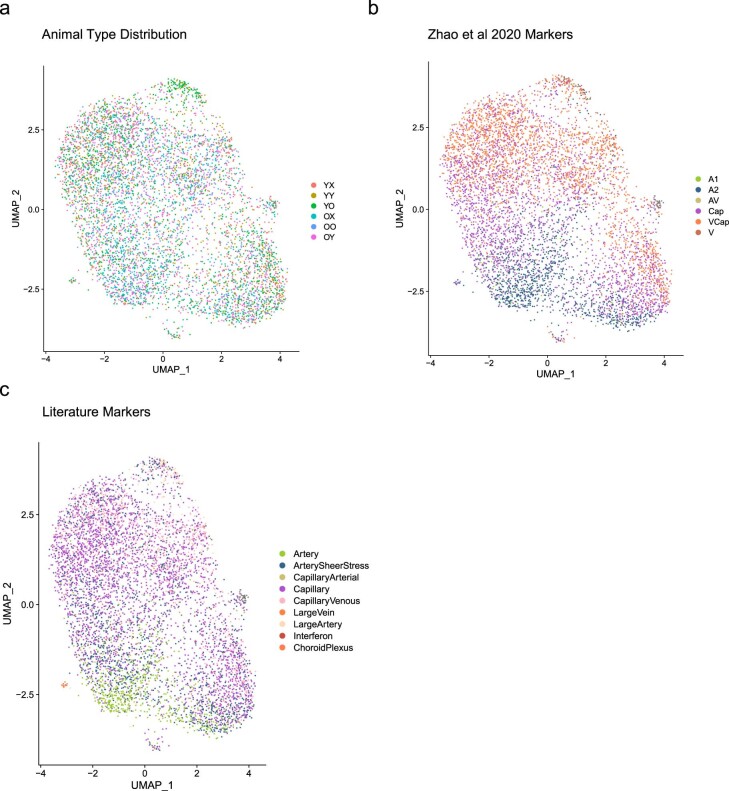
Animal type distribution and machine learning approaches to explore EC arteriovenous zonation. **a**. Animal type cell distribution across EC subclusters. **b-c**. Probabilistic programming cell class assignment using EC marker genes described by Zhao et al 2020^43^ (b) and others^41–43^ (c).

For further investigation, we performed high-resolution subclustering analysis to uncover the heterogeneity of these cell types. To reduce the effects of drastically different cell identities, we first grouped the identified cells into five distinct classes based on their expression profile, lineage, function and anatomical organization (Fig. 2). We delineated 75 distinct cell populations in accordance with the literature and our previous results^37^ (Fig. 2a–e). As with the major cell populations, all the identified subpopulations were represented in each animal type (Extended Data Fig. 6). For instance, we identified five subpopulations of ECs (Fig. 2f,g): ECs only expressing classic markers such as *Cldn5* (EC_1), which represent the largest fraction of ECs; ECs positive for astrocytic markers, such as *Slc1a3* (EC_2)^39^, potentially due to the presence of adherent RNA-containing astrocytic endfeet; ECs expressing the mitogenic/neovascularization marker *Lrg1* (ref. ^40^) (EC_3); ECs positive for the olfactory marker Omp (EC_4), possibly reflecting RNA in olfactory axons still attached to ECs; and ECs denoted by the expression of *Plvap*, known to be expressed in fenestrated ECs in the choroid plexus^41^ and circumventricular regions (EC_5). Although EC_2 has been characterized by others, EC_2 and EC_4 may be consequences of the mild dissociation protocol used (Fig. 2h). As found in our previous study^37^ and by others^41,42^, we did not observe distinct separation of arterial, capillary and venous ECs. However, select markers exhibited a zonation effect that further highlights the heterogeneity of ECs derived from different vascular beds (Extended Data Fig. 8). More specifically, as shown in Fig. 2i, probabilistic programming of cell class assignment using arterial/capillary/venous markers characterized in recent studies^41–43^ similarly displayed a clear zonation of ECs along the arteriovenous axis (Extended Data Fig. 8).

### Parabiosis reprograms the transcriptional landscape of brain cell types

Adult mouse tissues experience structural and functional improvement when exposed to young blood and deterioration when exposed to old blood. Our previous work detailed transcriptional changes that take place in brain cells during aging^37^, but not all these changes are necessarily of functional consequence. Heterochronic parabiosis might provide a first-pass filter to help identify those aging-related genes that drive the reversal of aging in the brain. We performed differential gene expression (DGE) analysis to identify the gene changes that are associated with the rejuvenation process in old heterochronic parabionts or with the aging acceleration process in young heterochronic parabionts. In the analysis, we attempted to compensate for the effects of the parabiosis surgery itself. We used a pseudobulk approach (Methods) to allow for chained pairwise comparisons of parabiosis-induced rejuvenation (RJV) or aging acceleration (AGA) with respect to the parabiosis surgery and the physiological aging process^44–46^. We also completed all relevant pairwise comparisons to directly compare the transcriptomes of any two animal types. Overall, our data allowed us to identify signatures that either shifted more toward reversal (RJV) or acceleration (AGA) of aging. More specifically, the RJV DGE dataset lists the old heterochronic genes (OY) taking into account isochronic mice (OO) and are associated with a transition to a ‘more youthful’ state with respect to normal aging (OX–YX). Conversely, the aging AGA DGE dataset lists the young heterochronic genes (YO) taking into account isochronic mice (YY) and are associated with a transition to a ‘more aged’ state with respect to normal aging (YX–OX) (Methods).

**Extended Data Fig. 9:**
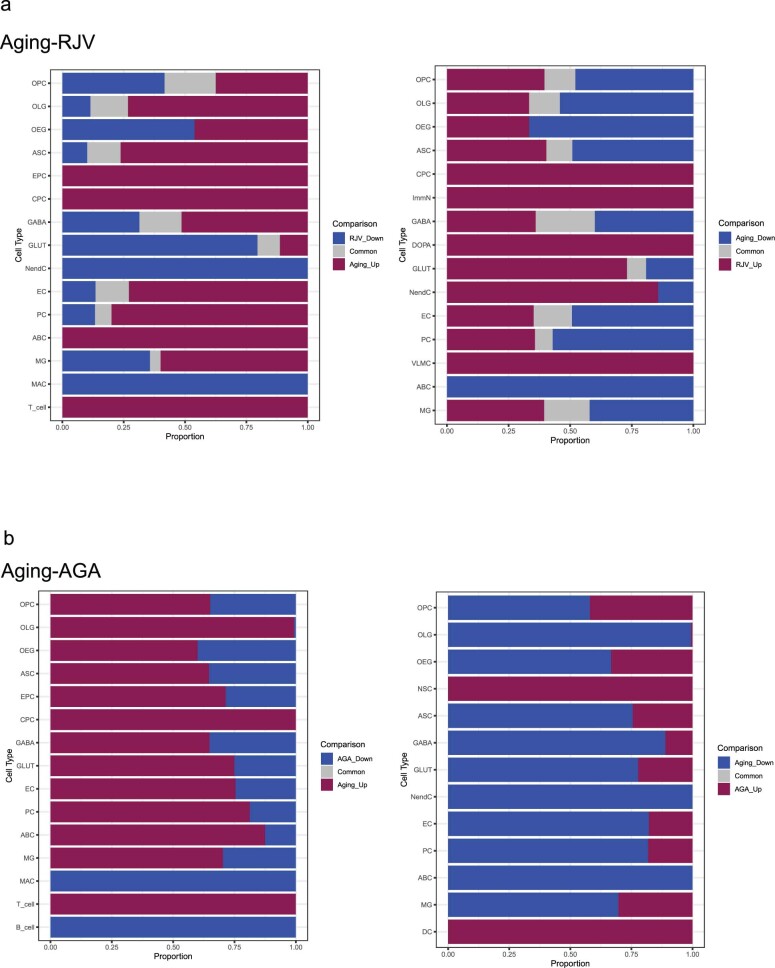
Composition of DGEs per cell type between Aging-RJV, and Aging-AGA. **a**. Bar graph of each cell type’s total FDR ≤ 0.05 DGEs split by logFC direction. The proportion of DGEs reflecting Aging and RJV is depicted, as well as the fraction of overlapping signatures (intersection in grey). **b**. Bar graph of each cell type’s total FDR ≤ 0.05 DGEs split by logFC direction. The proportion of DGEs reflecting Aging and AGA is depicted, as well as the fraction of overlapping signatures (intersection in grey).

Of the 20,905 total detected genes, 700 were significantly changed with aging in at least one cell type (false discovery rate (FDR) ≤ 0.05), whereas 442 were significantly changed in RJV and 155 in AGA (Supplementary Tables 3–11). As expected, we did not capture significant transcriptional changes in all the identified cell types, potentially due to the low number of cells sequenced in some populations and the low levels of detected transcription and dropouts inherent to scRNA-seq analysis^47^. None the less, our analysis also showed that the proportions of total DGEs per cluster that were common to aging and RJV varied from cell type to cell type, as did those that were common to aging and AGA (Extended Data Fig. 9). The DGEs in common between RJV and aging and AGA and aging, along with genes unique to each paradigm, demonstrate that not all effects of aging are reversed or accelerated by parabiosis and that heterochronic parabiosis may also work through genes and pathways independent of the aging process (Supplementary Tables 12 and 13).

**Fig. 3:**
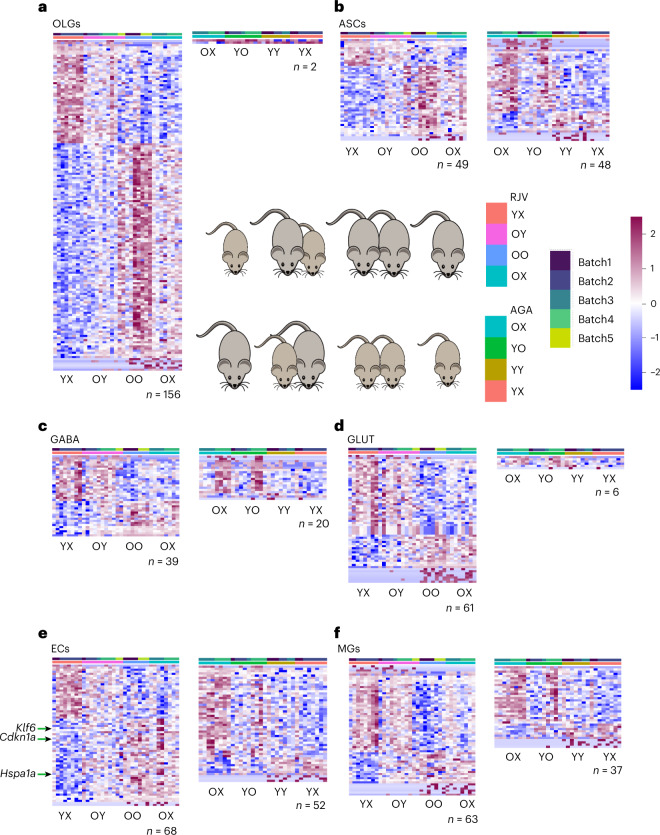
DGE across major cell types revealed RJV DGEs and aging AGA genes. **a**–**f**, An FDR ≤ 0.05 was used to identify significant DGE genes, with *n* denoting the total number of genes meeting this threshold. The RJV framework depicts normalized gene expression changes across YX, OY, OO and OX. The AGA framework depicts normalized gene expression changes across OX, YO, YY and YX. DGE genes are log_2_(*z*-scored) scaled across rows (all animals) and are ordered by descending log(FC), with OLGs (*n* = 156 RJV and *n* = 2 AGA) (**a**), ASCs (*n* = 49 RJV and *n* = 48 AGA) (**b**), GABA (*n* = 39 RJV and *n* = 20 AGA) (**c**), GLUT (*n* = 61 RJV and *n* = 6 AGA) (**d**), ECs (*n* = 68 RJV and *n* = 52 AGA) (**e**) and MGs (*n* = 63 RJV and *n* = 37 AGA) (**f**) in respective order. The color bar of the heatmap reflects the *z*-score, from negative (blue) to positive (magenta). The batch is denoted in the top annotation bar and animal type in the second annotation bar.

**Fig. 4:**
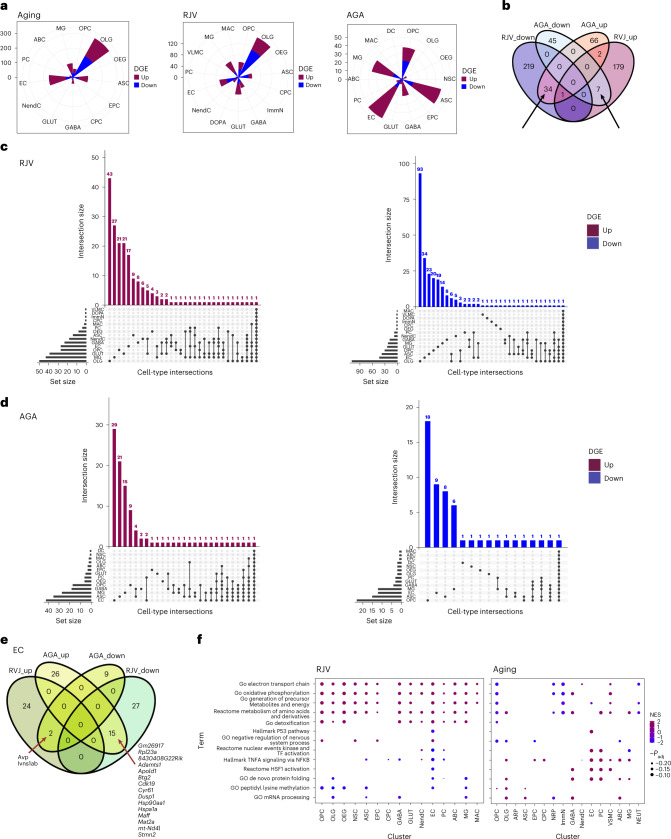
DGE characterization across cell types. **a**, Rose diagrams (circular histograms of number of DGEs) of aging, RJV and AGA across all cell types at FDR ≤ 0.05, colored by direction of log(FC) (up magenta, down blue). **b**, Venn diagram of RJV and AGA DGEs across all cell types, demonstrating bidirectional log(FC) changes between the comparisons (depicted with arrows). **c**,**d**, Upset plot of FDR = 0.05 DGE with positive log(FC) (upregulation) and negative log(FC) (downregulation) in both RJV (**c**) and AGA (**d**). The top bar height reflects the number of DGEs in the intersection (in common between the barbells below), and the side bar width reflects the magnitude of the set size. **e**, EC RJV and AGA DGE Venn diagram split by log(FC) sign, revealing genes that reverse direction between comparisons. The arrows point to listed bidirectional genes. **f**, GSEA dot plots (Benjamini–Hochberg-adjusted *P* value for multiple comparisons (*P*_adj_) ≤ 0.25) of representative terms across cell types in RJV and aging, with the size of dot proportional to inverse *P*_adj_ and color by NES from negative (blue) to positive (magenta).

On examination of the DGE datasets, a clear pattern of gene expression changes in RJV and AGA (FDR ≤ 0.05) was evident among our large cell populations (>5,000 cells) (Fig. 3 and Supplementary Tables 3–11), potentially due to higher statistical power. Specifically, oligodendrocytes (OLGs), astrocytes (ASCs), GLUT, GABA, ECs and microglia (MGs) yielded the largest number of putative DGEs (Figs. 3 and 4a and Supplementary Tables 3–11). Although OLGs had the largest number of cells, the number of DGEs normalized to the total number of OLGs in RJV, AGA and aging were 0.461, 0.00587 and 0.896, respectively. Conversely, ECs, an average cluster in size, had the third highest percentage of normalized RJV DGEs (1.0936) behind only NendCs (1.229) and GLUT (1.190), and had the highest percentage of AGA (0.836) and aging (2.638) DGEs. These data indicated that the EC population, most directly exposed to circulating factors, is highly affected in both the RJV and the AGA paradigms. This is in accordance with our previous observations showing improvements in the brain vasculature after heterochronic parabiosis^25^ and after growth differentiation factor (GDF)11 treatment^26^, and further emphasizes the key role that ECs play in regulating communication between blood and the brain^30^.

### Parabiosis shifts gene signatures associated with RJV and AGA

To find genes that are dysregulated across multiple cell types, we looked for key gene signatures that change in opposite directions in RJV and AGA. Across all cell types, a total of 41 unique genes was found to change their expression levels bidirectionally in RJV and AGA. Specifically, 34 genes were downregulated in RJV and upregulated in AGA (Fig. 4b and Supplementary Table 14). To identify cell types that change the direction of expression of the same genes in the parabiosis-induced RJV process and/or the parabiosis-induced AGA process, we created higher-dimensional Venn diagrams through upset plots (Fig. 4c,d). This analysis identified certain pairs of cell types that shared multiple DGEs in the same direction in RJV (such as ECs–MGs and OLGs–ASCs) and AGA (ECs–pericytes (PCs) and ECs–MGs) (Fig. 4c,d and Supplementary Tables 15 and 16). In multiple cell types in RJV, two major apolipoproteins (*Apoe* and *Clu*) were dysregulated, similar to findings in neurodegenerative diseases such as Alzheimer’s disease^48–50^ (Supplementary Tables 15 and 16).

**Fig. 5:**
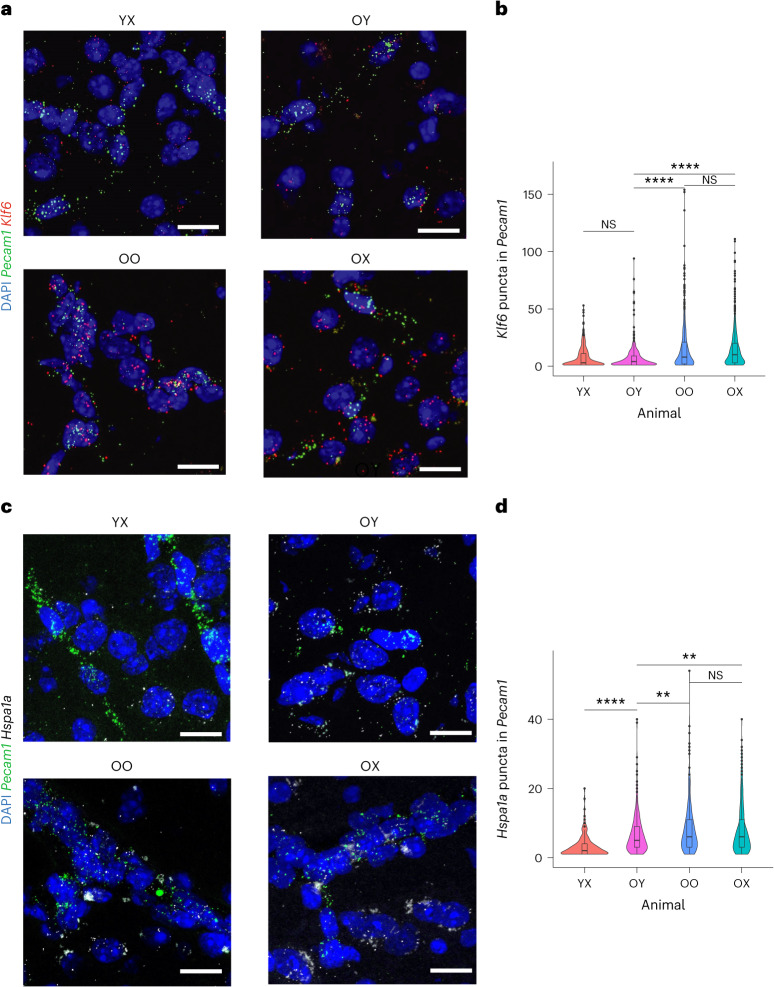
RNA in situ hybridization assays showing aging and RJV reversal of key aging-associated genes. **a**, Representative RNA images of mouse cortices showing *Klf6* puncta in *Pecam1*^+^ ECs in YX, OY, OO and OX mice. Scale bars, 20 µm. **b**, Violin and boxplot representation of RNA quantification (*n* = 3 biologically independent animals) by two-tailed Welch’s *t*-test with no multiple comparison adjustment for significance. *P* values for OY–YX: 0.473 (95% confidence interval (CI) −1.588, 0.737); OY–OO: 5.328 × 10^−19^ (95% CI −10.260, −6.620), OY–OX: 4.900 × 10^−28^ (95% CI −8.881, −6.244); OO–OX: 0.349 (95% CI −0.960, 2.714). Nonsignificant (NS) *P* > 0.05, ^*^P ≤ 0.05, ^**^P ≤ 0.01, ^***^P ≤ 0.001, ^****^P ≤ 0.0001. Boxplot minimum is the smallest value within 1.5× the IQR below the 25th percentile and maximum is the largest value within 1.5× the IQR above the 75th percentile. Boxplot center is the 50th percentile (median) and box bounds are the 25th and 75th percentiles. Outliers are >1.5× and <3× the IQR. **c**, Representative RNA images of mouse cortices showing *Hspa1a* puncta in *Pecam1*^+^ ECs in YX, OY, OO and OX mice. Scale bars, 20 µm. **d**, Violin and boxplot representation of RNA quantification (*n* = 4 biologically independent animals) by two-tailed Welch’s *t*-test with no multiple comparison adjustment for significance. *P* values for OY–YX: 3.535 × 10^−16^ (95% CI 2.727, 4.373); OY–OO 0.008 (95% CI −2.603, −0.385); OY–OX: 0.006 (95% CI −2.177, −0.372); OO–OX: 0.686 (95% CI −0.847, 1.286). NS *P* > 0.05, ^*^P ≤ 0.05, ^**^P ≤ 0.01, ^***^P ≤ 0.001, ^****^P ≤ 0.0001. Boxplot minimum is the smallest value within 1.5× the IQR below the 25th percentile and maximum is the largest value within 1.5× the IQR above the 75th percentile. Boxplot center is the 50th percentile (median) and box bounds are the 25th and 75th percentiles. Outliers are >1.5× and <3× the IQR. Source data

ECs again demonstrated the greatest number of genes that change direction of expression (log(fold-change) (log(FC))) between RJV and AGA, further highlighting their susceptibility to the aging process and their potential for manipulation. Overall, a substantial percentage of genes with expression that was increased with aging decreased their ECs after heterochronic parabiosis. For example, the transcription factor (TF) *Maff*, which is highly upregulated with aging in ECs, was found to be downregulated in RJV and upregulated in AGA (Fig. 4e and Supplementary Table 14). Similarly, the aging-upregulated genes *Hsp90aa1* and *Hspa1a*, which encode heat shock response proteins, *Adamsts1*, which is induced on shear stress^51^, *Apold1*, which is responsive to hypoxia/ischemia^52^ and lipopolysaccharide treatment^30^, *Cyr61*, which encodes an extracellular matrix protein involved in angiogenesis^53^, and *Dusp1*, which participates in EC migration^54^ along with *Stmn2*, a gene involved in the microtubule organization^37,39^, were all downregulated in RJV and upregulated in AGA (Fig. 4e and Supplementary Table 14). There were only two genes with expression that changed in the opposite direction—down with aging and up after heterochronic parabiosis. One is *Avp*, a hormone that has been known for some time to signal to brain ECs, which, however, were not known to secrete it. The other is *Ivns1ab*, known to stabilize the cytoskeleton in some cell types and protect from cell death induction due to actin destabilization^55^ (Fig. 4e and Supplementary Table 14). The DGE analysis also showed that the TF *Klf6*, which was one of the most upregulated genes with aging in ECs, was differentially downregulated in RJV, as was its downstream target *Smad7* (Supplementary Tables 3 and 4). Considering that *Klf6* expression is known to be regulated by vascular injury^56,57^, we further characterized its transcriptional changes by RNA in situ hybridization. As shown in Fig. 5a,b, *Klf6* expression was indeed found to be highly upregulated with aging in ECs (*Pecam1*^*+*^). Heterochronic parabiosis reversed this change in the old parabionts, bringing its transcriptional levels of *Klf6* close to those seen in young ECs (Fig. 5b).

Taken together, our computational analysis identified numerous aging-related genes across multiple cell types, including ECs, with expression restored in old mice and/or disrupted in young mice after heterochronic parabiosis, suggesting their potential involvement in the rejuvenation and/or aging acceleration process, respectively. These data are again consistent with the notion that parabiosis is likely to act in part by regulating processes important to vascular structure and health.

### Heterochronic parabiosis reverses aging-induced pathways

We next performed an implementation of gene set enrichment analysis (GSEA)^58^ to reveal biological processes and molecular pathways associated with aging, RJV and AGA. Composite ranks were calculated for each gene based on FDR and log(FC). As with our previous study on old and young animals^37^, the ranking metric used yielded many significant terms for most cell types (Supplementary Table 17). Specifically, in the RJV model, the most gene sets were observed in oligodendrocyte precursor cells (OPCs), OLGs, olfactory ensheathing glia (OEGs), ASCs, GABA, GLUT, NendCs, ECs and MGs, whereas, in the AGA model, only OPCs, OLGs and PCs yielded significant terms. This further reinforced our observation that heterochronic parabiosis induces stronger aging-related gene signature changes in the old parabionts.

Among the key ontologies that were downregulated in aging and upregulated in RJV across multiple cell types were pathways related to mitochondrial activity, such as gene ontology (GO) oxidative phosphorylation, and GO electron transport chain, as well as oxidative stress homeostasis and metabolism pathways, such as GO detoxification, GO generation of precursor metabolites and energy and reactome metabolism of amino acids, and derivatives followed this trend (Fig. 4f and Supplementary Table 17). RJV-downregulated pathways such as GO DNA conformation change and GO peptidyl lysine methylation further demonstrated that the epigenetic machinery is functionally perturbed in various cell populations with parabiosis (Fig. 4f and Supplementary Table 17).

In addition to the above ontologies, ECs displayed a clear pattern of normalized enrichment score (NES) sign reversal between aging and RJV, corresponding to our DGE profiling. Changes in mitochondrial and metabolic pathways were found in RJV. Processes that were downregulated include inflammatory pathways such as hallmark tumor necrosis factor α (TNFA) signaling via nuclear factor-κB (NF-κB), and apoptosis and the senescence-associated hallmark P53 pathway. Likewise, proteostasis-associated pathways, such as Reactome HSF1 activation and GO de novo protein folding, were found to be upregulated with aging and downregulated in RJV (Fig. 4f and Supplementary Table 17). Collectively, these data suggested that heterochronic parabiosis changes the metabolic profile, improves proteostatic machinery and reduces aging-associated apoptosis or senescence to improve EC function, consistent with recent findings in aortic ECs^59^.

We then focused on further exploring the decline of proteostasis, an acknowledged hallmark of aging, in ECs. Our pathway analysis revealed upregulation of several stress-inducible pathways in ECs that are presumably activated in response to misfolded protein accumulation in aging and suppressed in RJV (Fig. 4f and Supplementary Table 17). For example, we examined the gene expression levels of *Hspa1a*, which encodes a stress-inducible heat shock protein. At the DGE level, as mentioned above, *Hspa1a* was found to be upregulated in aging and downregulated in RJV in ECs (Supplementary Tables 3 and 4). To verify this change, we performed RNA in situ hybridization. As shown in Fig. 5c,d, we detected a significant decline in the number *Hspa1a* puncta expressed by ECs in heterochronic parabionts.

### Parabiosis activates global remodeling of GRNs

**Fig. 6:**
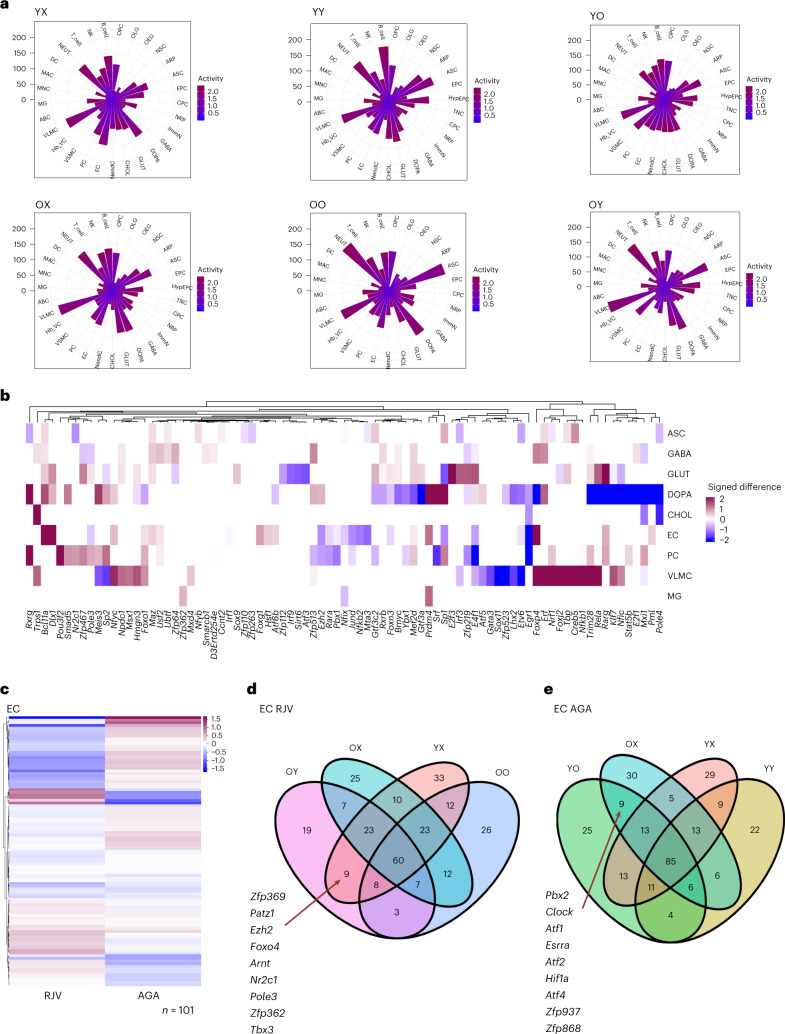
Gene regulatory analysis reveals transcriptionally active cell types and GRN reversals between RJV and AGA. **a**, Rose diagrams of GRN scores per cell type across YX, YY, YO, OX, OO and OY. The color scale from blue to magenta reflects the degree of GRN activity (Methods). **b**, Difference heatmap of active GRN TFs corresponding to RJV/AGA log(FC) change sign. Magnitude is the absolute magnitude of the difference, and direction is positive for upregulation in RJV (magenta) and negative for upregulation in AGA (blue). **c**, Heatmap of EC-active GRN TFs plotted by RJV and AGA log(FC), with upregulation magenta, downregulation blue, clustered with Euclidean distance, average linkage. **d**,**e**, Venn diagrams of EC RJV (**d**) and AGA (**e**) animal frameworks’ active GRN TFs. The arrows point to those TFs in common between OY and YX and YO and OX, respectively.

In an attempt to identify key regulatory TFs involved in RJV or AGA independent of changes in their own expression levels, we utilized the SCENIC approach to detect gene regulatory networks (GRNs) comprising TFs and their downstream effector genes^60^. Each animal type was profiled individually to identify putatively active GRNs, stratified by cell class. The young unpaired and young isochronic animals had fewer cell types with higher frequencies (>100) of higher (>1) GRN activity scores than young heterochronic animals (Fig. 6a). Vascular smooth muscle cells (VSMCs), a recently identified perivascular-like cell type of the brain vasculature^39^, frequently had high activity across all animal types (Fig. 6a). In old heterochronic animals, vascular leptomeningeal cells (VLMCs), NEUT, ASCs and DOPA had higher frequencies of high (>1) GRN activity scores than old unpaired animals (Fig. 6a and Supplementary Table 18). These putative changes in GRN activity may suggest an increase in global remodeling in the old and young heterochronic mice to reflect RJV and AGA, respectively.

RJV via parabiosis has a distinct transcriptional landscape that follows the opposite direction of expression in AGA. The signed absolute difference in log(FC) identifies TFs that are bidirectional between RJV and AGA, meaning that they have increased expression in RJV and downregulation in AGA, or vice versa. DOPA demonstrated the most extreme instances of TFs that change direction, followed by VLMCs, then ECs (Fig. 6b and Supplementary Table 14). Considering that ECs displayed many bidirectional TFs, we further explored their entire transcriptional landscape. Of the EC TFs in old and young heterochronic brains, most demonstrated opposite regulation in RJV and AGA (Fig. 6c). TFs in common between old heterochronic and young unpaired ECs that reflect RJV included essential regulators of EC function such as *Tbx3* (refs. ^61,62^) and *Foxo4* (ref. ^63^), a member of the FOXO family of TFs that are components of a fundamental aging regulatory pathway*, Patz1*, which has been implicated in regulating p53 levels and senescence in ECs^64^, and *Arnt*, which participates in aryl hydrocarbon receptor signaling and is involved in several aspects of vascular biology^65^ (Fig. 6d). Likewise, young heterochronic and old unpaired ECs that reflect the AGA construct shared the hypoxia response genes *Atf1*, *Atf2* and *Hif1a*, indicating a stressed EC profile, as well as *Atf4* which has been implicated in angiogenesis^66^ (Fig. 6e).

### Parabiosis alters intercellular communication networks

**Fig. 7:**
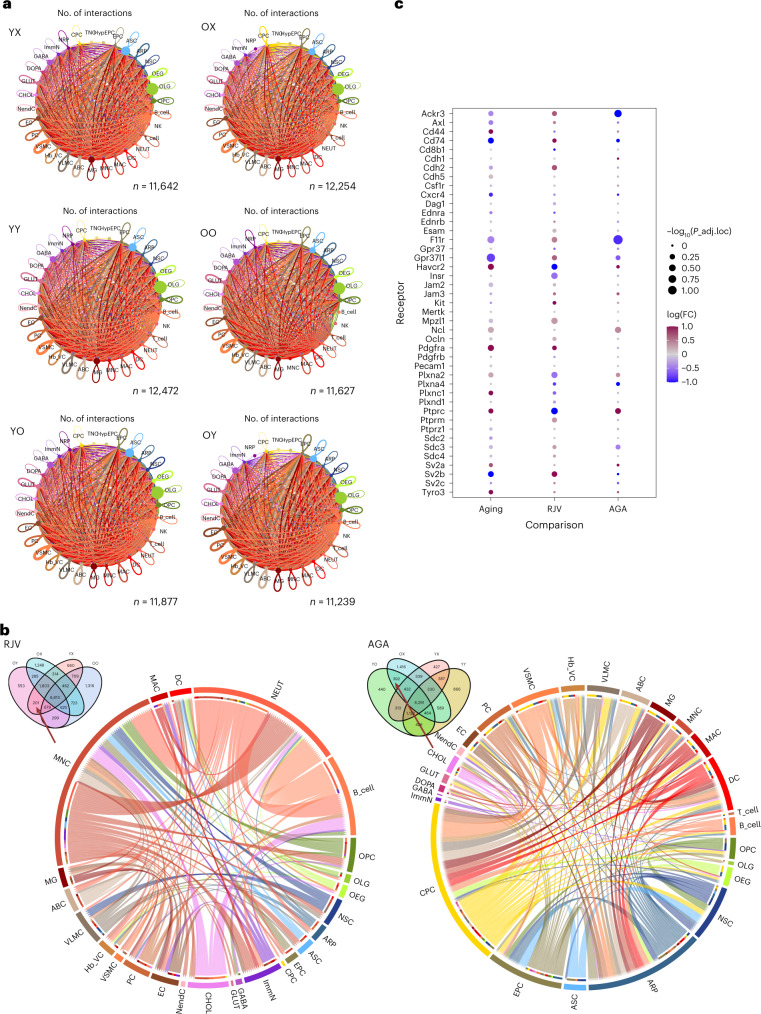
Cell–cell communication is affected by aging and parabiosis. **a**, Summarization network graphs of the number of ligand–receptor interactions between cell types in YX, YY, YO, OX, OO and OY mice. Node size is proportional to cell population size. Edge width and transparency of color are proportional to the number of all edges between a set of nodes. **b**, Chord diagrams representing the informatically predicted unique source:target:receptor:ligand pairings identified only in the rejuvenation model of OY and YX (Venn diagram inset, left panel) or the aging acceleration model of YO and OX (Venn diagram inset, right panel). **c**, For all identified EC receptors, edgeR DGE QLF test metrics are shown for the aging, RJV and AGA paradigms. Node size is inversely proportional to the Benjamini–Hochberg-adjusted *P* value for multiple comparisons and node color is scaled by intensity of log(FC) from blue (negative, downregulation) to magenta (positive, upregulation).

Altered intercellular communication is one of the hallmarks of aging^1^. Although many studies have examined the actions of blood-borne factors on CNS cells, few have looked at factors secreted by the CNS cells themselves and how they are modified by aging. The importance of such secreted factors has been shown to be dysregulated in inflammation and degeneration^67–70^. To analyze changes in intercellular communication within the brain we used CellChat^71^. We first measured the total number of interactions for each animal type to elucidate the number of cell–cell communication connections (Fig. 7a and Supplementary Table 19). We found that the old heterochronic parabionts exhibited fewer putative connections than the old unpaired and old isochronic animals, whereas the young heterochronic parabionts exhibited more putative connections than in the young unpaired and young isochronic animals (Fig. 7a). In RJV, we discovered connections triggered by VLMCs and ABCs, both of which act physiologically as barrier cells. For example, we found that VLMCs and ABCs potentially signaled to ASCs, ECs and MGs, whereas they received signals from neural stem cells (NSCs), neuronal-restricted precursors (NRPs) and VSMCs (Fig. 7b and Supplementary Table 19). Conversely, in AGA, more signaling was triggered by CPCs. Specifically, CPCs signaled to OPCs and various neuronal cell types (DOPA, GLUT, cholinergic neurons (CHOL)), whereas they received signals from VSMCs and GLUT. In this paradigm, we also observed more signaling triggered by EPCs, which are cells that also act physiologically as barriers because they form the epithelial lining of the ventricles (Fig. 7b and Supplementary Table 19). Taken together, this computational analysis highlighted the prominent roles for various barrier cells in the brain parenchyma, as well as CPCs, in processes accompanying or mediating the effects of parabiosis.

**Extended Data Fig. 10:**
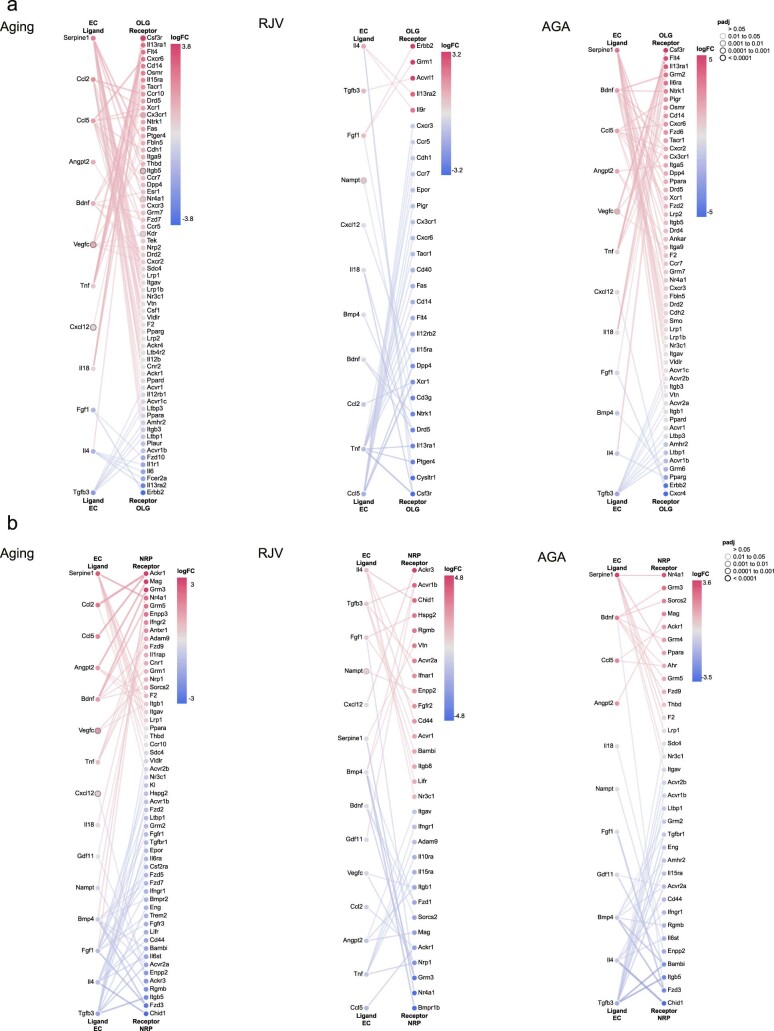
Intercellular communication networks between EC-OLG and EC-NRP revealed aging-related interactions that were modified by heterochronic parabiosis. Canonical EC ligands and their cognate receptors in OLG (a) or in NRP (b) are shown in each paradigm (Aging, RJV, AGA). In all panels of ligand-receptor interactions, node color represents the magnitude of the DGE (logFC as estimated by DGE) such that the most significantly up-regulated genes are in magenta, and the downregulated genes are in blue. Node borders indicate multiple testing corrected Benjamini-Hochberg FDR for statistical significance of DGE as calculated by edgeR. Edge color represents the sum of scaled differential expression magnitudes from each contributing node, while width and transparency are determined by the magnitude of the scaled differential expression (see details in the Methods section).

Following our previously published approach^37^, we pursued ligand–receptor interactions determined by encapsulating FDR and log(FC) as the metric for putative association. This computational analysis showed that there are certain aging-induced changes in cell–cell communication that were reversed in the old heterochronic parabionts and/or potentiated in the young heterochronic parabionts. As an example, we detailed interactions involving EC ligands, which are known to secrete signals that regulate neurogenesis^72^ in NRPs and, recently, were reported to be implicated in signaling networks with OLG lineage cells^73^. We delineated a clear pattern of connection direction reversal between aging and RJV for most ligands, as well as between RJV and AGA, with AGA exhibiting a direction similar to aging (Extended Data Fig. 10). Specifically, we identified several secreted factors that could mediate these intercellular relationships, including cytokines/inflammatory mediators such as CXCL12 (ref. ^74^) and growth factors such as brain-derived neurotrophic factor^75^. Another way of investigating the data is to look for aging/RJV-dependent changes in the levels of cellular receptors. We applied this analysis to ECs, known to be highly influenced by factors found in blood and recognized to be subject to interactions with PCs and ASCs. The genes encoding EC receptors found in the cell–cell communication analysis are shown in Fig. 7c, demonstrating the log(FC) reversal between aging and RJV, and same log(FC) direction between aging and AGA, in many receptors.

Our computational analysis identified numerous cell–cell communication networks that are perturbed during the aging process and modified on heterochronic parabiosis, and highlighted the significance of ECs as a potential target for therapeutics, because its intercellular interactions are affected by both aging and heterochronic parabiosis.

### Heterochronic parabiosis regulates the senescence state

**Fig. 8:**
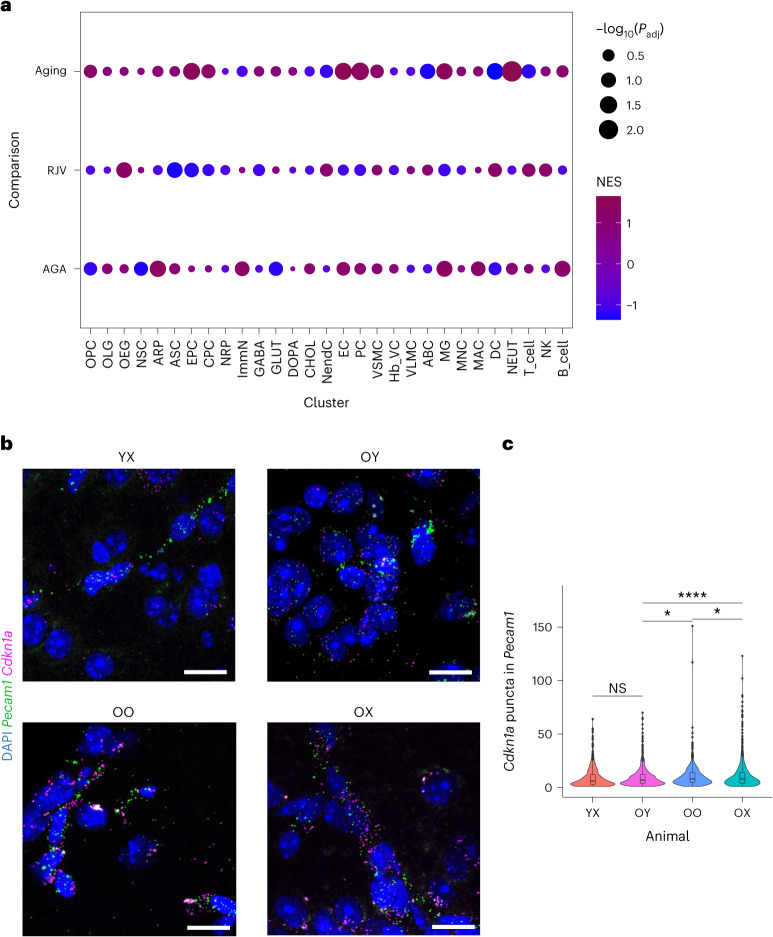
Senescence status demonstrated shifts in aging and RJV. **a**, Dot-plot representation of senescence-associated marker genes curated from the literature^76,78^, permuted against each cell type in aging, RJV and AGA with fast GSEA. The inverse log_10_(*P*_adj_) values for multiple comparisons (Benjamini–Hochberg) reflect the size of the dot and NESs reflect color from blue (negative enrichment) to magenta (positive enrichment). **b**, Representative RNA in situ images of mouse cortices showing *Cdkn1a* puncta in *Pecam1*^+^ ECs in YX, OY, OO and OX mice. Scale bars, 20 µm. **c**, Violin and boxplot representation of RNA quantification (*n* = 6 biologically independent animals) by two-tailed Welch’s *t*-test with no multiple comparison adjustment for significance. *P* values: OY–YX: 0.810 (95% CI −0.930, 1.190); OY–OO: 0.0484 (95% CI −1.670, −0.006); OY–OX: 0.0000874 (95% CI −3.063, −1.025); and OO–OX: 0.0251 (95% CI −2.260, −0.151). NS *P* > 0.05, ^*^P ≤ 0.05, ^**^P ≤ 0.01, ^***^P ≤ 0.001 and ^****^P < 0.0001. Boxplot minimum is the smallest value within 1.5× the IQR below the 25th percentile and maximum is the largest value within 1.5× the IQR above the 75th percentile. Boxplot center is the 50th percentile (median) and box bounds are the 25th and 75th percentiles. Outliers are >1.5× and <3× the IQR. Source data

The effects of parabiosis on cellular senescence are beginning to be recognized^76,77^. To explore this in more detail, we performed an implementation of GSEA with a reference gene set of literature-defined, senescence-associated genes (Supplementary Table 20)^76,78^ against the preranked aging, RJV and AGA gene sets to determine functional enrichment of senescence in these paradigms, and their directions. Across 20 of the 29 examined cell populations, we observed directionality reversal between aging and RJV based on NES (Fig. 8a and Supplementary Table 21). In the vast majority of cell types, aging was associated with increased senescence consistent with previous studies^79^, whereas RJV resulted in reduced senescence. The NES direction was the same in aging and AGA for 20 cell types, indicative of recapitulation of the aging process in AGA (Fig. 8a and Supplementary Table 21). To confirm the effects of parabiosis on senescence, we performed RNA in situ hybridization to evaluate the expression levels of *Cdkn1a*, a well-known senescence-associated gene. Specifically, we observed a significant decrease in *Cdkn1a* expression in ECs (*Pecam1*^*+*^) in the old heterochronic parabiotic brains compared with the old isochronic and old brains (Fig. 8b-c).

Our computational analysis indicates that heterochronic parabiosis modulates the senescence signature in multiple brain cell types and suggests the possibility that the cellular senescence regulation itself may contribute, at least partly, to the positive and negative effects of parabiosis in the brains of old and young parabionts, respectively.

## Discussion

Over the past few years, many studies have demonstrated that aging is a highly dynamic and malleable process, with several types of treatment reported to rejuvenate tissues and organs and, in turn, extend organismal healthspan and lifespan^80,81^. Among these interventions, heterochronic parabiosis appears to be one of the most effective. Despite this, the mechanism(s) by which parabiosis acts at the cellular and molecular levels to improve tissue function remains elusive. In the present study, we present a comprehensive single-cell survey of the gene expression changes that occur in the aged and young mouse brain after heterochronic parabiosis as a step toward understanding how parabiosis improves brain function.

We first assessed the cellular complexity of the parabiotic brains and showed that cell identity and composition were largely retained. Thus, functional changes associated with parabiosis are probably due, at least substantially, to changes in the gene expression profiles of CNS cells. A limitation of the present study is that, as tissue dissociation is an inherent part of the single-cell sequencing workflow, this might have resulted in nonuniform sampling problems^82^. Therefore, we cannot completely rule out shifts in the numbers of certain cell populations^22,25^. Future studies employing single-nuclei sequencing approaches^83^ and spatially resolved transcriptomics^84,85^ may shed more light on this matter.

We further explored the gene expression changes associated with aging and heterochronic parabiosis in all identified brain cell types to discover genes and pathways that are implicated in the rejuvenation and aging acceleration. To this end, we identified the primary cell types exhibiting these changes and revealed the common and cell-type-specific, aging-induced signatures and transcriptional programs that were rescued after parabiosis in the old brains and/or disrupted in the young brains. As corroborated by recent reports, our data provide evidence that heterochronic parabiosis effectively modulates multiple manifestations of canonical aging hallmarks, including altered intercellular communication^69^, loss of proteostasis^86^, defects in mitochondrial dysfunction^59,87–89^ and cellular senescence^76,77,90–92^.

Our analyses suggest that the modulation of the aging process is mediated by reprogramming of the associated transcriptional signatures across multiple brain cell types. In support of this notion, aging-induced changes in the epigenetic status of the aged mouse brain^93^, as well as of the aged liver and blood tissues^94^, were recently reported to be reversed after heterochronic parabiosis. The reprogramming of the transcriptome presented in the present study is somewhat similar to the reprogramming recently reported to take place in rats subjected to caloric restriction^95^. These findings raise the exciting possibility that both interventions could promote tissue rejuvenation by mitigating the appearance of similar aging-associated epigenetic alterations, and consequently their induced transcriptional changes. Thus, future studies investigating the exact epigenetic regulators and mechanisms that are responsible for these types of changes will be of high importance^81,96^.

Parabiosis is a complex process and stimulates a number of changes—positive and negative—in both parabionts^97^. It is interesting that it can be argued that the positive effects of young blood factor exposure can overcome not only aging-driven changes in gene expression, but also processes such as systemic inflammation and stress stimulated by the parabiosis surgery itself^14,98^. Our data support the view that parabiosis also exerts effects independent of the aging process, an aspect incorporated into our analysis. Moreover, parabiosis-driven effects may be mediated through means other than reversing the effects of normal aging. In recognition of this possibility, we designated the DGEs from the chained pairwise comparisons that have no overlap with aging DGEs (Supplementary Tables 12 and 13).

One of the primary discoveries of our study is that the EC transcriptome is dynamically regulated by both aging and heterochronic parabiosis. We found that ECs, when compared with other brain cell types, exhibited one of the highest fractions of aging-related genes that were rescued after heterochronic parabiosis in the old brain and, similarly, the highest fraction of aging-related genes that were disrupted after heterochronic parabiosis in the young brain. This finding supports our previous research that the vasculature is strongly affected by aging and disease and is capable of regrowth after heterochronic parabiosis^25^ or systemic GDF11 treatment^26^. We observed that a subset of ECs was classified as mitogenic, expressing high levels of *Lrg1* and *Lcn2* (Fig. 2f,g), and it is reasonable to speculate that the growth of these cells, the growth of which is probably prevented or suspended by the inflammatory environment of the aged brain, may be among the cell populations that respond to these interventions. Although proteostasis in brain ECs has not been thoroughly investigated, they are apparently long-lived cells^30,99^ and, like neurons, might therefore accumulate protein aggregates with age^100^, potentially compromising their function. As previously shown, ECs become senescent with age^101,102^, but parabiosis may reverse that phenotype as well. Taken together, these findings underline the strong susceptibility and malleability of ECs, which are directly exposed to secreted factors in both brain parenchyma and blood, to adapt to changes in their microenvironment, which is consistent with pervious observations from our lab^25,26^ and others^30,43,59,69^. Therefore, ECs, despite comprising <5% of the total number of brain cells^103^, are a promising and accessible target for the treatment of aging and its associated diseases^104^.

Collectively, our computational matrices and web portals provide unprecedented data that can be further explored to form working hypotheses for future studies. Similar to other recent reports^89,105,106^, our study advances fundamental understanding of the mechanisms underlying the aging process and potential interventions that go beyond descriptive studies of cell states. Our work extends current knowledge about the effects of heterochronic parabiosis on the aging process and supports the key role of the brain vasculature in mediating these effects. Effectively, in one, albeit complex procedure, parabiosis improves many of the individual processes, such as mitochondrial dysfunction, proteostasis collapse and cellular senescence, which are usually targeted separately by therapeutic interventions. Based on data in this article, future work aimed at developing therapeutics might focus on individual processes in specific cell types, on regulating levels of regulatory factors secreted from within the CNS or even on regulating the transcriptional states of cells in the brain.

## Methods

### Animals

C57BL/6J inbred male mice (JAX no. 000664; CD45.1^−^CD45.2^+^) and B6.SJL-Ptprc^a^ Pepc^b^/BoyJ male mice (JAX no. 002014; CD45.1^+^CD45.2^−^) were housed in the Harvard Biolabs Animal Facility under standard conditions. All experimental procedures were approved in advance by the Institutional Animal Care and Use Committee of Harvard University (AEP no. 10-23) and are in compliance with federal and state laws. On the day of sacrifice, young mice were aged 3–4 months (13–15 weeks) and old mice were 20–22 months (80–87 weeks), analogous to human ages 18–20 and 65–70 years, respectively^107^.

### Parabiosis

Parabiosis surgeries were performed as previously described^25,108^ with a few modifications. In brief, mice were sedated with controlled isoflurane anesthesia and placed on heating pads to prevent hypothermia. Ophthalmic ointment was applied to prevent dryness of the eyes. The lateral sides of the mice were then carefully shaved and aseptically prepared. Matched skin incisions were made to the shaved sides and the knee and elbow joints were tied together with nonabsorbable sutures (Reli no. SK7772) to facilitate coordinated movement. Surgical wound clips (BD Autoclips 9 mm, no. 427631) and absorbable sutures (Ethicon no. J385H) were then used to join the skins together. On surgery, parabionts were injected subcutaneously with the anti-inflammatory enrofloxacin (5 mg kg^−1^) to prevent infection and the analgesics carprofen (10 mg kg^−1^) and buprenorphine (0.1 mg kg^−1^) to manage pain (single injection every 12 h for up to 3 d post-surgery). In each injection, 0.5 ml of prewarmed 0.9% (w:v) sodium chloride was also provided to prevent dehydration. Pairs were then kept in clean cages and placed on to heating pads for up to 24 post-surgeries to maintain body temperature. Throughout the surgery and postoperative recovery, each pair was monitored continuously and potential signs of pain and distress were recorded, although several physical characteristics were also analyzed, including pair weights. After 15 d post-surgery, pairs were sedated again briefly to allow removal of the surgical wound clips and remnants of the absorbable sutures. Parabiotic pairs were maintained for 4–5 weeks (mean 31.5 d; Extended Data Fig. 2) before processing for tissue collection and subsequent analysis.

### Blood chimerism analysis

To evaluate blood crosscirculation we followed the same approach as previously described^25^, in which the presence in both partners of heterochronic pairs of blood cells bearing congenic markers from both the aged (CD45.2^+^) and the young (CD45.1^+^) parabionts is demonstrated. Specifically, in the heterochronic pairs we used young mice carrying the congenic marker CD45.1 (JAX no. 002014) and old mice carrying the congenic marker CD45.2 (JAX no. 000664), whereas in the young isochronic pairs we used mice carrying either CD45.1 or CD45.2. For the blood chimerism analysis, spleens were extracted from the mice and single cells were mechanically isolated by passing the spleen through a 40-μm filter. Erythrocytes were lysed with ACK lysis buffer (Thermo Fisher Scientific, catalog no. A1049201) for 3 min on ice and single cells were resuspended in Hanks Balanced Salt Solution (Thermo Fisher Scientific, catalog no. 14025-134) containing 2% fetal bovine serum. Splenocytes were then filtered through a 40-µm filter and stained with an antibody cocktail (Pacific Blue anti-CD45.1 (BioLegend, catalog no. 110722; 1:100 dilution), APC anti-CD45.2 (BioLegend, catalog no. 109814; 1:100 dilution), PE anti-TER-119 (Thermo Fisher Scientific, catalog no. 12-5921-82; 1:200)) and the fixable viability dye Zombie Aqua (BioLegend, catalog no. 423101; 1:300 dilution) for 30 min on ice in the dark. Cells were then washed and fixed in 1% paraformaldehyde before analysis. Cells were gated on physical parameters to identify singlets followed by gating on the Zombie Aqua^low^TER-119^−^ population to identify live nonerythroid cells. These cells were subsequently gated as CD45.1^+^ or CD45.2^+^ to measure the frequency of donor-derived blood cells from one partner in the spleen of the other partner. Flow cytometry analysis was performed using a BD LSR II flow cytometer (BD Biosciences) and data were analyzed with FlowJo software (v.10). We found that the partner-derived cells represented 30–50% of splenocytes (mean 41.3%; Extended Data Fig. 1), consistent with successful establishment of parabiotic crosscirculation^38^. This analysis could not be applied to old isochronic mice, because old mice carrying the congenic marker CD45.1 were not available for purchase; however, the crosscirculation in old isochronic mice has been well characterized previously^23^.

### Brain tissue dissociation

Brain tissue collection was performed at the same time of day (9–10am), processing one pair of mice per day, thus limiting circadian variation^109^. Brain tissue dissociation was performed as previously described^37^. Briefly, mice were CO_2_ anesthetized and then rapidly decapitated. Brains were extracted and hindbrain regions removed. The remaining tissue was mechanically and enzymatically dissociated into single cells and kept on ice for no longer than 1 h until further processing.

### ScRNA-seq

For the scRNA-seq experiments, 8 YX, 9 YO, 9 YY, 8 OX, 11 OY and 12 OO brains were analyzed, with two animals (one pair) sacrificed per day as mentioned above. Briefly, after dissociation, cells were diluted in ice-cold phosphate-buffered saline (PBS) containing 0.4% bovine serum albumin at a density of 1,000 cells µl^−1^. For every sample, 17,400 cells were loaded into a Chromium Single Cell 3′ Chip (10x Genomics) and processed following the manufacturer’s instructions. ScRNA-seq libraries were prepared using the Chromium v.2 Single Cell 3′ Library and Gel Bead kit v.2 and i7 Mutiplex kit (10x Genomics). Libraries were pooled based on their molar concentrations. Pooled libraries were then loaded at 2.07 pM and sequenced on a NextSeq 500 instrument (Illumina) with 26 bases for read1, 57 bases for read2 and 8 bases for Index1. Cell Ranger (v.2.0) (10x Genomics) was used to perform sample de-multiplexing, barcode processing and single-cell gene unique molecular identifier (UMI) counting, whereas a digital expression matrix was obtained for each experiment with default parameters, mapped to the 10x reference for mm10, v.1.2.0. After the initial sequencing, the samples in each pool were re-pooled based on the actual number of cells detected by Cell Ranger (Extended Data Fig. 2a), aiming to sequence each sample to a similar depth (number of reads per cell) (mean 43,107 reads per cell; Extended Data Fig. 3c). Multiple NextSeq runs were conducted to achieve >70% sequencing saturation as determined again by Cell Ranger (median: 75%).

### Raw data processing and quality control for cell inclusion

Basic processing and visualization of the scRNA-seq data were performed using the Seurat package (v.3.2.1.9002)^110^ in R (v.3.6.1). The initial dataset contained 158,767 cells with data for 21,876 genes (Extended Data Fig. 3) The average number of UMI (nCount_RNA) and nonzero genes (nFeature_RNA) are 2828.298 and 1206.153, respectively. The data were log(normalized) and scaled to 10,000 transcripts per cell. Variable genes were identified with FindVariableFeatures() function with the following parameters to set minimum and maximum average expressions and minimum dispersion: mean.cutoff(0.00125, 3), dispersion.cutoff(1,Inf). Next, principal component analysis (PCA) was carried out and the top 50 PCs were stored. Clusters were identified with FindNeighbors() by constructing a K-nearest neighbor (KNN) graph and clustered with the Louvain algorithm with FindClusters() at resolution 2, represented by Uniform Manifold Approximation and Projection (UMAP). All clusters with only one cell were removed and clusters with >8% mitochondrial genes, clusters with min nCount_RNA <1,000 and clusters with min nFeature_RNA 500 were flagged for exclusion, resulting in 80 initial clusters. Animals with low average number of genes >0 (<700), percentage mitochondria >1.5 and not having cell contribution to each cluster were assessed for exclusion. In total, five isochronic old and one isochronic young were removed from the dataset (to retain eight YX, seven YY, nine YO, eight OX, seven OO and eleven OY animals), and the above clustering steps were performed at resolution 2. For quality control (QC) filtering, we selectively removed clusters with minimum percentage mitochondria 0, maximum percentage mitochondria 5%, min_nFeature_RNA 250, max_nFeature_RNA 6000, min_nCount_RNA 200, max_nCount_RNA 30000, min_cells=5. After the second round of QC, we retained 130,889 cells and 20,905 genes. The average nCount_RNA, nonzero genes, percentage mitochondrial RNA and percentage ribosomal RNA were 2736.187, 1368.007, 1.149 and 5.135, respectively. We re-clustered at resolution 2 to identify 69 clusters. The final preprocessing step was to remove probable doublet artifacts arising from the cocapture of multiple cells in one droplet. After an initial round of cluster identity determination as assessed in the next section, we employed a doublet-finding technique by searching for the top differential markers of each identified cluster/subcluster with the FindMarkers() function, and marked doublets/multiplets as any cluster in which >40% of its cells express seven of the top ten genes specific to an initially identified cell type and any other outside of the class of the cell type with which it is associated. These clusters were removed from the downstream analysis and clustering was again performed at resolution 2, representing 105,329 cells with similar retention to other studies^39,111^ and 69 clusters across 20,905 genes. We examined the UMAP space and all clusters are represented by all batches, so no further correction was warranted (Extended Data Fig. 4a,b).

### Determination of cell-type identity

We used multiple cell-specific/enriched gene markers that have been previously described in the literature to assist in determining cell-type identity^37^. We identified 31 major cell types with distinct expression profiles: OPCs, OLGs, OEGs, NSCs, astrocyte-restricted precursors (ARPs), ASCs, EPCs, hypendymal cells (HypEPCs), tanycytes (TNCs), CPCs, NRPs, immature neurons (ImmNs), GABA, DOPA, GLUT, CHOL, NendCs^37^, ECs, PCs, VSMCs, hemoglobin-expressing vascular cells (Hb-VCs)^37^, VLMCs, ABCs, MGs, monocytes (MNCs), macrophages (MACs), dendritic cells (DCs), NEUT, T cells (T_cell), natural killer (NK) cells and B cells (B_cell) (Fig. 1c and Supplementary Table 1).

We then arranged all the identified cell types based on their expression profile, lineage, function and topology into five classes of cells (OLG lineage and OEGs, astroependymal cells and NSCs, neuronal lineage, vasculature cells and immune cells). For each group, we re-clustered the subcategorized cell types using top 50 PCs at resolution 5. The annotation of the subclusters was performed similar to the identification of the main cell clusters.

### DGE analysis

After initial QC preprocessing and determination of cellular identities, we utilized the muscat package (v.1.0.0) in R (v.3.6.1) to perform pseudobulk DGE analysis with edgeR’s quasi-likelihood F (QLF) test^44–46^. Seurat objects were exported to SingleCellExperiments and reads were collapsed per animal to ‘sum’ based on ‘counts’. The ‘rejuvenation framework’ RJV follows the design contrast (OY–OX)–(OO–YX), assigning in the design matrix: OY: 1, OO: −1; OX: −1; YX: 1. The ‘aging acceleration framework’ AGA follows the design contrast (YO–YX)–(YY–OX), assigning in the design matrix: YO: 1; YY: −1; YX: −1; OX: 1. Pairwise comparisons for OXvYX, OYvOX, OYvOO, OOvOX, YOvYX, YOvYY and YYvYX were also computed. Via muscat, edgeR generates a log(FC), log(counts per min), *F*, p_val (*P* value), p_adj.loc and p_adj.glb. We used the Benjamini–Hochberg-adjusted *q* value p_val.loc in all downstream thresholding. Our ability to establish a baseline level of transcription is reliant on the number of cells measured and thus larger clusters’ variation can be more adequately modeled. HypEPCs and TNCs did not contain enough cells over multiple animals to successfully derive statistics. For all mouse types, raw normalized transcript per million (TPM) values were calculated and percentage of expression per animal type. For heatmap representations, the log_2_(*z*-score) of each animal’s TPM in the rejuvenation or aging acceleration process was calculated gene-wise (by row).

### Pathway analysis

GSEA was performed with the fgsea R package (v.1.12.0)^112^. Using the protocol previously implemented^113^, for each cell population and DGE comparison, genes were ranked by multiplying −log_10_(p_val) with the sign of the log(FC) and converted to *Homo sapiens* orthologs using biomaRt (v.2.46.2). From MSigDB, we used five gene sets: Hallmark pathways, GO biological process, Kyoto Encyclopedia of Genes and Genomes (KEGG), BioCarta and Reactome. In fgsea, 1,000 permutations were performed with minimum gene set size of 15 and maximum 500. Gene sets with FDR ≤ 0.25 were considered significantly enriched. Term annotations and grouping of those overrepresenting the same pathway were derived from Cytoscape software (v.3.5.1) and the AutoAnnotate app (v.1.2) as previously described^37^. The NES directionality was used to collate cell-type pathways per DGE comparison. Dot-plot representations are a composite of FDR and NES.

### GRNs

We employed SCENIC to assess GRNs and score their activity, using the R implementation (v.1.1.2-2)^60^. Each animal type was analyzed with respect to each lineage. Briefly, we used GENIE3 to identify genes that are coexpressed with TFs. Then, RCisTarget prunes these coexpression modules to create GRNs (regulons). The direct targets of each TF are found using *cis*-regulatory motif analysis. AUCell scores each regulon’s activity, binarized to on/off at threshold 0.7. The regulon activity per animal type per lineage is graphed with rose diagram histograms. Regulons with TFs that change log(FC) direction between RJV and AGA are identified and those regulons changing direction in at least eight cell clusters are further investigated. The magnitude of the log(FC) difference between RJV and AGA can take the form of the absolute difference, with the sign of the difference positive if rejuvenation is >0 and aging acceleration is <0, negative if aging acceleration is >0, and rejuvenation is <0 or zeroed out if they are in the same direction. Regulon matrices of each animal type across all clusters, along with row-wise regulon count, are reported.

### Cell–cell communication

Cell–cell communication between cell types per animal type was assessed using the CellChat tool^71^. The number of interaction graphs per animal were thresholded at interactions reaching *P* ≤ 0.05 and graphed with netVisual_circle. Rejuvenation-associated construct graphs were the subset of unique receptor:ligand:source:target combinations of interactions occurring only in OY and YX, and aging-associated construct graphs were the same combination occurring only in YO and OX, graphed via the circlize package (0.4.13.1001)^114^. EC receptors in all six animal types were collapsed into a master list, with DGE *P*_adj_/log(FC) graphed via ggplot2. EC receptor graphs per animal type were constructed via CellChat netVisual_aggregate.

Cell–cell communication per comparison was also conducted as previously described^37^ using the CCInx package (0.5.1). Per-comparison plots were generated between ligand–receptor pairs using the CCInx tool.

### Cellular senescence analysis

Cellular senescence was investigated using functional enrichment on preranked genes against known senescence marker genes as described in the literature^76,78^. Briefly, the preranked (−log_10_(p*_*val)) multiplied by the sign of the log(FC) aging, RJV and AGA gene lists were permuted 1,000× against the gene set using the fgsea algorithm implemented in ClusterProfiler (v.3.14.3). The NES and *P*_adj_ (Benjamini–Hochberg) are reported.

### EC class assignment

EC ‘zonation’ was assessed through deep learning using the CellAssign framework^115^ (v.0.99.21, tensorflow_2.2.0.9000). Gene markers from Zhao et al.^43^ and other sources^41–43^ were used to define arterial–venous–capillary markers. The learning rate used was 1 × 10^−2^, with a min_delta of 0.25 and 10 runs on a V100 graphics processing unit hosted on the FAS Cannon cluster.

### RNA in situ hybridization

RNA in situ hybridization was performed on fresh-frozen brain tissue from at least three mice for each relevant condition (YX, OY, OO, OX). For sample preparation, mice were sacrificed via cervical dislocation and the brains were rapidly extracted and embedded in optimal cutting temperature (OCT; Tissue Tek) on dry ice, and subsequently stored at −80 °C until further processing. Brains were divided into 14-μm cryostat sections and RNA in situ hybridizations were carried out using the RNA in situ Multiplex Fluorescent Manual Assay kit (Advanced Cell Diagnostics (ACD)) per the manufacturer’s instructions. Briefly, thawed sections were fixed in 4% paraformaldehyde in PBS and dehydrated in sequential incubations with ethanol, followed by a 30-min Protease IV treatment and washing in 1× PBS. Appropriate combinations of hybridization probes were incubated on tissue for 2 h at 40 °C, followed by four amplification steps. Sections were subsequently stained with DAPI and mounted with Prolong Gold mounting medium (Thermo Fisher Scientific, catalog no. P36930). Brain regions were selected based on areas of high expression levels of assessed examined genes, according to the Allen Brain Atlas^116^. Commercially available and validated probes for *Cdkn1a* (ACD, catalog no. 408551), *Hspa1a* (ACD, catalog no. 488351), *Klf6* (ACD, catalog no. 426901) and *Pecam1* (ACD, catalog no. 316721) were utilized per the manufacturer’s instructions. For each mouse and tissue, three Bregma-matched sections were imaged. Images (four per tissue section) were acquired with a Zeiss LSM 880 Confocal Microscope with identical settings across sections and represented as maximum intensity projections of acquired confocal *z*-stacks. Analysis was done using a script within CellProfiler software (v.4.2.1), in which *Cdkn1a*, *Hspa1a* or *Klf6* puncta with a diameter between 1 and 12 pixels located within the perinuclear space (100 pixels of DAPI-positive nuclei) were identified and quantified. Cells with two or more *Pecam1*^+^ puncta were designated *Pecam1*^+^ ECs. For *Hspa1a* and *Cdkn1a* experiments, the EC marker *Pecam1* was labeled by fluorophore Atto 647, whereas target probes were labeled by Atto 488 (*Hspa1a*) and Atto 550 (*Cdkn1a*). For *Klf6* experiments, *Pecam1* was labeled by the fluorophore Alexa Fluor-488 whereas *Klf6* was labeled by the fluorophore Atto 550. Lipofuscin granules largely associated with aged brain tissue were avoided utilizing the 1- to 12-pixel cutoff for identifying puncta. For each animal, an unstained tissue was imaged as a negative control and to assess levels of background fluorescence.

### Statistics and reproducibility

No statistical methods were used to predetermine sample sizes; our samples sizes were determined iteratively. No randomization was performed. Data collection and analysis were not performed blind to the conditions of the experiments. Animals with low average number of genes >0 (<700), percentage mitochondria >1.5 and not having cell contribution to each cluster were assessed for exclusion. In total, five OO and one YY were removed from the dataset. Further, clusters of poor quality, over percentage mitochondria = 5%, under nFeature_RNA = 250, over nFeature_RNA = 6,000, under nCount_RNA = 200, over nCount_RNA = 30,000, fewer than 5 cells were removed (see Methods for full details). All statistical analyses were performed with R (v.3.6.1). To generate *P* values for cell counts, ANOVA was conducted between animal types per cell type (rstatix 0.6.0). For validation of gene expression changes by RNA in situ hybridization, two-tailed Welch’s *t*-test was conducted as indicated (rstatix 0.6.0). Data distribution was assumed to be normal with equal variance, but this was not formally tested.

### Reporting summary

Further information on research design is available in the Nature Portfolio Reporting Summary linked to this article.

## Supplementary information

Reporting Summary

Supplementary Table 1 List of abbreviations for all cell types and subpopulations and major markers delineating them. The cell type abbreviations and their major markers are listed in the first tab (Cell_Type_Abbreviations_Markers). In the second tab (Cell Type Seurat Markers), the top ten (by avg_log2FC) informatically derived markers from Seurat’s FindAllMarkers() for each cell type are listed, along with Wilcoxon’s rank-sum *P* value, Bonferonni-adjusted *P* value (p_val_adj), average log_2_(FC) (avg_log2FC), pct.1 (the percentage of cells in that cell type expressing the gene) and pct.2 (the percentage of cells in all other cell types expressing the gene).

Supplementary Table 2 Metrics of pairwise comparison of cell types by cell number. For each pairwise comparison, per cell type, ANOVA was applied (one tailed) with no multiple comparison correction, with significance designation (NS *P* > 0.05, ^*^P ≤ 0.05, ^**^P ≤ 0.01, ^***^P ≤ 0.001).

Supplementary Table 3 DGE metrics of RJV, per cell type. edgeR/muscat metrics from the QLF test were computed for each cluster in a comparison, with log(FC), *P* value, Benjamini–Hochberg *P*-value adjustment for multiple comparisons (p_adj.loc is per cluster, p_adj.global is with respect to all clusters in class) reported. TPM values (‘tpm’) for each animal type, per cell type, are listed. Percentage expression (‘pct’) of each gene for each animal type, per cell type, is listed.

Supplementary Table 4 DGE metrics of AGA, per cell type. edgeR/muscat metrics from the QLF test were computed for each cluster in a comparison, with log(FC), *P* value, Benjamini–Hochberg *P*-value adjustment for multiple comparisons (p_adj.loc is per cluster, p_adj.global is with respect to all clusters in class) reported. TPM values (‘tpm’) for each animal type, per cell type, are listed. Percentage expression (“pct”) of each gene for each animal type, per cell type are listed.

Supplementary Table 5 DGE metrics of aging, per cell type. edgeR/muscat metrics from the QLF test were computed for each cluster in a comparison, with log(FC), *P* value, Benjamini–Hochberg *P*-value adjustment for multiple comparisons (p_adj.loc is per cluster, p_adj.global is with respect to all clusters in class) reported. TTPM values (‘tpm’) for each animal type, per cell type, are listed. Percentage expression (‘pct’) of each gene for each animal type, per cell type are listed.

Supplementary Table 6 DGE metrics of OYvOO, per cell type. edgeR/muscat metrics from the QLF test were computed for each cluster in a comparison, with log(FC), *P* value, Benjamini–Hochberg *P*-value adjustment for multiple comparisons (p_adj.loc is per cluster, p_adj.global is with respect to all clusters in class) reported. TPM values (‘tpm’) for each animal type, per cell type, are listed. Percentage expression (‘pct’) of each gene for each animal type, per cell type are listed.

Supplementary Table 7 DGE metrics of OYvOX, per cell type. edgeR/muscat metrics from the QLF test were computed for each cluster in a comparison, with logFC, *P* value, Benjamini–Hochberg *P* value adjustment for multiple comparisons (p_adj.loc is per cluster, p_adj.global is with respect to all clusters in class) reported. TPM values (“tpm”) for each animal type, per cell type are listed. Percentage expression (‘pct’) of each gene for each animal type, per cell type are listed.

Supplementary Table 8 DGE metrics of OOvOX, per cell type. edgeR/muscat metrics from the QLF test were computed for each cluster in a comparison, with log(FC),*P* value, Benjamini–Hochberg *P*-value adjustment for multiple comparisons (p_adj.loc is per cluster, p_adj.global is with respect to all clusters in class) reported. TTPM values (‘tpm’) for each animal type, per cell type, are listed. Percentage expression (‘pct’) of each gene for each animal type, per cell type are listed.

Supplementary Table 9 DGE metrics of YOvYY per cell type. edgeR/muscat metrics from the QLF test were computed for each cluster in a comparison, with log(FC), *P* value, Benjamini–Hochberg *P*-value adjustment for multiple comparisons (p_adj.loc is per cluster, p_adj.global is with respect to all clusters in class) reported. TPM values (‘tpm’) for each animal type, per cell type, are listed. Percentage expression (‘pct’) of each gene for each animal type, per cell type are listed.

Supplementary Table 10 DGE metrics of YOvYY, per cell type. edgeR/muscat metrics from the QLF test were computed for each cluster in a comparison, with log(FC), *P* value, Benjamini–Hochberg *P*-value adjustment for multiple comparisons (p_adj.loc is per cluster, p_adj.global is with respect to all clusters in class) reported. TPM values (‘tpm’) for each animal type, per cell type, are listed. Percentage expression (‘pct’) of each gene for each animal type, per cell type are listed.

Supplementary Table 11 DGE metrics of YYvYX per cell type. edgeR/muscat metrics from the QLF test were computed for each cluster in a comparison, with log(FC), *P* value, Benjamini–Hochberg *P*-value adjustment for multiple comparisons (p_adj.loc is per cluster, p_adj.global is with respect to all clusters in class) reported. TPM values (‘tpm’) for each animal type, per cell type are listed. Percentage expression (‘pct’) of each gene for each animal type, per cell type are listed.

Supplementary Table 12 RJV and aging common DGEs and unique DGEs, per cell type. DGE FDR ≤ 0.05 genes common between RJV and aging, but also those only found in RJV and those only found in aging are listed per cell type.

Supplementary Table 13 AGA and aging common DGEs and unique DGEs, per cell type. DGE FDR ≤ 0.05 genes common between AGA and aging, but also those only found in AGA, and those only found in aging are listed per cell type.

Supplementary Table 14 DGE bidirectional DGEs between RJV and AGA. For RJV and AGA DGE genes FDR ≤ 0.05 per cell type, report the genes that are RJV up (log(FC) > 0) and AGA down (log(FC) <0), or RJV down (log(FC) < 0) and AGA up (log(FC) > 0). Count is the number of times a gene is bidirectionally expressed across cell types.

Supplementary Table 15 Matrices of all DGE FDR ≤ 0.05 log(FC) values across cell types. Per comparison, per gene, clusters where the gene’s significance is FDR ≤ 0.05 have their log(FC) value reported. ‘Up’ column is the sum of clusters with log(FC) > 0, ‘Down’ column is the sum of clusters with log(FC) < 0.

Supplementary Table 16 DGE logFC values across all cell types. Per comparison, per gene, collated log(FC) values across all clusters reporting DGE, with no thresholding. ‘Up’ column is the sum of clusters with log(FC) > 0. ‘Down’ column is the sum of clusters with log(FC) < 0.

Supplementary Table 17 Matrices of all identified significant GSEA terms per comparison across cell types. Fast preranked GSEA (fGSEA) Benjamini–Hochberg-adjusted *P* value for multiple comparisons ≤0.25 significant terms are collated per comparison across all cell types by NES. Pathway and process metaclasses are described in Methods. ‘Up’ column is the sum of cell types with NES > 0. ‘Down’ column is the sum of cell types with NES < 0.

Supplementary Table 18 SCENIC regulon matrices per animal type. Per animal type, per cell type, SCENIC regulon activity scores are reported. Column ‘counts’ is the sum of cell type that have a regulon score.

Supplementary Table 19 Cell–cell communication networks in RJV, AGA and per animal type*.* For RJV, the set of unique source:target:receptor:ligand pairs that are found only in OY and YX combined. For AGA, the set of unique source:target:receptor:ligand pairs that are found only in YO and OX combined. YX, YY, YO, OX, OO and OY display the CellChat^71^ networks derived for each animal type (see details in Methods). ‘Source’ is the cell type the ligand comes from, whereas ‘Target’ is the cell type found matching the ligand’s receptor. Probability and *P* value are the statistical measures derived by CellChat. Ligand–receptor pairs are given interaction names and assigned to a pathway. Annotation provides the type of interaction are secreted signaling, ECM–receptor, cell–cell contact. Evidence codes and relevant PubMed IDs are provided by CellChat.

Supplementary Table 20 Literature-curated senescence-associated genes. Senescence-associated genes were curated from the literature^76,78^, for use as a reference gene set to perform fGSEA. HGNC.symbol denotes *Homo sapiens* gene symbol, MGI.ID denotes MGI ID number, MGI.symbol is *Mus musculus* gene symbol, Name is the long name of the gene and Feature type denotes the type of gene or pseudogene.

Supplementary Table 21 Senescence status GSEA per comparison. For RJV, AGA and aging, fGSEA via ClusterProfiler was run against a literature-curated senescence gene set (Supplementary Table 20) to derive enrichment score, NES, *P* value, Benjamini–Hochberg-adjusted *P* value for multiple testing correction, rank and genes in the leading edge.

## Source data

Source Data Fig. 5 Raw images of RNA in situ hybridization for Klf6 (a) YX.

Source Data Fig. 5 Raw images of RNA in situ hybridization for Klf6 (a) OY.

Source Data Fig. 5 Raw images of RNA in situ hybridization for *Klf6* (a) OO.

Source Data Fig. 5 Raw images of RNA in situ hybridization for *Klf6* (a) OX.

Source Data Fig. 5 Raw images of RNA in situ hybridization for *Hspa1a* (c) YX.

Source Data Fig. 5 Raw images of RNA in situ hybridization for *Hspa1a* (c) OY.

Source Data Fig. 5 Raw images of RNA in situ hybridization for *Hspa1a* (c) OO.

Source Data Fig. 5 Raw images of RNA in situ hybridization for *Hspa1a* (c) OX.

Source Data Fig. 8 Raw images of RNA in situ hybridization for *Cdkn1a* YX.

Source Data Fig. 8 Raw images of RNA in situ hybridization for *Cdkn1a* OY.

Source Data Fig. 8 Raw images of RNA in situ hybridization for *Cdkn1a* OO.

Source Data Fig. 8 Raw images of RNA in situ hybridization for *Cdkn1a* OX.

## Data Availability

Raw data are available on the Gene Expression Omnibus under accession no. GSE222510. Data exploration of this scRNA-seq study is currently available at https://rubinlab.connect.hms.harvard.edu/parabiosis and on the Broad Single Cell Portal at https://singlecell.broadinstitute.org/single_cell/study/SCP2011/aging-mouse-brain-parabiosis. Source data are provided with this paper.

## Code availability

Code is available upon request: https://github.com/kmh005/rubin_parabiosis.
