# Author Correction: Heterochronic parabiosis reprograms the mouse brain transcriptome by shifting aging signatures in multiple cell types

**DOI:** 10.1038/s43587-025-00804-6

**Published:** 2025-01-29

**Authors:** Methodios Ximerakis, Kristina M. Holton, Richard M. Giadone, Ceren Ozek, Monika Saxena, Samara Santiago, Xian Adiconis, Danielle Dionne, Lan Nguyen, Kavya M. Shah, Jill M. Goldstein, Caterina Gasperini, Ioannis A. Gampierakis, Scott L. Lipnick, Sean K. Simmons, Sean M. Buchanan, Amy J. Wagers, Aviv Regev, Joshua Z. Levin, Lee L. Rubin

**Affiliations:** 1 Department of Stem Cell and Regenerative Biology, Harvard University, Cambridge, MA USA; 2 Harvard Stem Cell Institute, Cambridge, MA USA; 3 Stanley Center for Psychiatric Research, Broad Institute of MIT and Harvard, Cambridge, MA USA; 4 Klarman Cell Observatory, Broad Institute of MIT and Harvard, Cambridge, MA USA; 5 Joslin Diabetes Center, Boston, MA USA; 6 Paul F. Glenn Center for the Biology of Aging, Harvard Medical School, Boston, MA USA; 7 Howard Hughes Medical Institute, Koch Institute of Integrative Cancer Research, Department of Biology, Massachusetts Institute of Technology, Cambridge, MA USA

**Keywords:** Neural ageing, Neuro-vascular interactions, Blood-brain barrier, Alzheimer's disease, Gene regulatory networks

Correction to: *Nature Aging* 10.1038/s43587-023-00373-6, published online 9 March 2023.

Following publication, the authors were alerted to a potential duplication in two of the micrographs shown in the paper. After a detailed review, it appears that two probes (Hspa1a and Pecam1) in an RNA in situ hybridization experiment were unintentionally swapped in the representative images shown in Figs. 5c and 8b of the published manuscript. Upon revisiting the original data files, the authors determined that this error arose during the preparation of representative images for the final resubmission. Specifically, raw microscopic images were prepared in Fiji, which applies a default labeling system (C1, C2, C3 etc.). Unfortunately, in the raw image files (.czi), Hspa1a and Pecam1 were labeled as C3 and C1, respectively, leading to an inadvertent switch.

The authors have now replaced Figs. 5c and 8b with the correct Pecam1/Hspa1a and Pecam1/Cdkn1a probes, respectively. Moreover, the color of the signals in these images have been changed to avoid any additional confusion. The figures are updated in the HTML and PDF versions of the article.

In the Supplementary Data accompanying this amendment, the authors include the erroneous and corrected representative images for comparison. Importantly, these micrographs were utilized only for representative image purposes and do not affect any conclusions in the paper.

## Supplementary information

Supplementary Data

