# Supplementary material for: Heterochronic parabiosis reprograms the mouse brain transcriptome by shifting aging signatures in multiple cell types
Source: Nat Aging. 2023 Mar 9;3(3):327–45. doi: 10.1038/s43587-023-00373-6 (PMC10154248; doi:10.1038/s43587-023-00373-6)
Supplement: Supplementary file 1 — Reporting Summary [file 43587_2023_373_MOESM1_ESM.pdf]

nature portfolio

Corresponding author(s): Lee L. Rubin, Methodios Ximerakis

Last updated by author(s): Jan 23, 2023

Reporting Summary

Nature Portfolio wishes to improve the reproducibility of the work that we publish. This form provides structure for consistency and transparency in reporting. For further information on Nature Portfolio policies, see our Editorial Policies and the Editorial Policy Checklist.

Statistics

For all statistical analyses, confirm that the following items are present in the figure legend, table legend, main text, or Methods section.

n/a	Confirmed	
☐	☒	The exact sample size (*n*) for each experimental group/condition, given as a discrete number and unit of measurement
☐	☒	A statement on whether measurements were taken from distinct samples or whether the same sample was measured repeatedly
☐	☒	The statistical test(s) used AND whether they are one- or two-sided<br>*Only common tests should be described solely by name; describe more complex techniques in the Methods section.*
☒	☐	A description of all covariates tested
☐	☒	A description of any assumptions or corrections, such as tests of normality and adjustment for multiple comparisons
☐	☒	A full description of the statistical parameters including central tendency (e.g. means) or other basic estimates (e.g. regression coefficient) AND variation (e.g. standard deviation) or associated estimates of uncertainty (e.g. confidence intervals)
☐	☒	For null hypothesis testing, the test statistic (e.g. *F*, *t*, *r*) with confidence intervals, effect sizes, degrees of freedom and *P* value noted<br>*Give P values as exact values whenever suitable.*
☒	☐	For Bayesian analysis, information on the choice of priors and Markov chain Monte Carlo settings
☒	☐	For hierarchical and complex designs, identification of the appropriate level for tests and full reporting of outcomes
☒	☐	Estimates of effect sizes (e.g. Cohen's *d*, Pearson's *r*), indicating how they were calculated

Our web collection on statistics for biologists contains articles on many of the points above.

Software and code

Policy information about availability of computer code

Data collection	Cell Ranger (version 2.0
Data analysis	R (version 3.6.1)<br>Seurat package (version 3.2.1.9002}<br>muscat package (version 1.0.0)<br>fgsea package (version 1.12.0)<br>biomaRt package (version 2.46.2}<br>SCENIC package (version 1.1.2-2)<br>CellChat package (version 1.0.0)<br>circlize package (0.4.13.1001}<br>CClnx package (0.5.1)<br>ClusterProfiler package (version 3.14.3)<br>CellAssign package (version 0.99.21, tensorflow_2.2.0.9000}<br>rstatix package (version 0.6.0)<br>Cytoscape software (version 3.5.1)<br>AutoAnnotate app (versionl.2)<br>CellProfiler (version 4.2.1)<br>FlowJo software (version 10)

For manuscripts utilizing custom algorithms or software that are central to the research but not yet described in published literature, software must be made available to editors and reviewers. We strongly encourage code deposition in a community repository (e.g. GitHub). See the Nature Portfolio guidelines for submitting code & software for further information.

Data

Policy information about availability of data

All manuscripts must include a data availability statement. This statement should provide the following information, where applicable:

- Accession codes, unique identifiers, or web links for publicly available datasets
- A description of any restrictions on data availability
- For clinical datasets or third party data, please ensure that the statement adheres to our policy

Data is available in GEO under accession:
GSE222510 (https://www.ncbi.nlm.nih.gov/geo/query/acc.cgi?acc=GSE222510)
in the Broad Single Cell Portal:
https://singlecell.broadinstitute.org/single_cell/study/SCP2011/
and in our RShiny Application:
https://rubinlab.connect.hms.harvard.edu/parabiosis/

Human research participants

Policy information about studies involving human research participants and Sex and Gender in Research.

Reporting on sex and gender	There were no human subjects in this experiment.
Population characteristics	There were no human subjects in this experiment.
Recruitment	There were no human subjects in this experiment.
Ethics oversight	There were no human subjects in this experiment.

Note that full information on the approval of the study protocol must also be provided in the manuscript.

Field-specific reporting

Please select the one below that is the best fit for your research. If you are not sure, read the appropriate sections before making your selection.

☒ Life sciences ☐ Behavioural & social sciences ☐ Ecological, evolutionary & environmental sciences

For a reference copy of the document with all sections, see nature.com/documents/nr-reporting-summary-flat.pdf

Life sciences study design

All studies must disclose on these points even when the disclosure is negative.

Sample size	scRNA-seq was conducted on 56 animals (16 unpaired and 40 parabionts). No statistical methods were used to predetermine sample sizes; our sample sizes were determined iteratively.
Data exclusions	Data from 6 parabionts were excluded for poor quality scRNA-seq reads (including low average number of genes above 0 (<700), percentage mitochondria > 1.5, and not having cell contribution to each cluster). Clusters of poor quality over percent mitochondria=5%, under nFeature_RNA=250, over nFeature_RNA=6000, under nCount_RNA=200, over nCount_RNA=30000, less than 5 cells were removed.
Replication	Parabiosis is a lengthy and costly procedure. We provided an ample sample size with which to make statistical inferences.
Randomization	Mice were unpaired young, unpaired old, isochronic parabiosis old, isochronic parabiosis young, or from the young or old heterochronic parabiosis pairs. No randomization was performed, but animals were distributed evenly among 5 scRNA-seq batches.
Blinding	There was no blinding, given the nature of the parabiosis procedure.

Reporting for specific materials, systems and methods

We require information from authors about some types of materials, experimental systems and methods used in many studies. Here, indicate whether each material, system or method listed is relevant to your study. If you are not sure if a list item applies to your research, read the appropriate section before selecting a response.

Materials & experimental systems

n/a	Involved in the study
☐	☒ Antibodies
☒	☐ Eukaryotic cell lines
☒	☐ Palaeontology and archaeology
☐	☒ Animals and other organisms
☒	☐ Clinical data
☒	☐ Dual use research of concern

Methods

n/a	Involved in the study
☒	☐ ChIP-seq
☐	☒ Flow cytometry
☒	☐ MRI-based neuroimaging

Antibodies

Antibodies used	Pacific Blue anti-CD45.1 (Biolegend no. 110722)<br>https://www.biolegend.com/en-us/products/pacific-blue-anti-mouse-cd45-1-antibody-3105<br><br>APC anti-CD45.2 (Biolegend no. 109814)<br>https://www.biolegend.com/en-gb/products/apc-anti-mouse-cd45-2-antibody-2759<br><br>PE anti-TER-119 (Thermo Fisher Scientific, no. 12-5921-82)<br>https://www.thermofisher.com/antibody/product/TER-119-Antibody-clone-TER-119-Monoclonal/12-5921-82<br><br>Zombie Aqua (Biolegend no. 423101)<br>https://www.biolegend.com/en-us/products/zombie-aqua-fixable-viability-kit-8444?GroupID=BLG2181
Validation	All the above antibodies are well characterized commercial antibodies. For each one, the specificity has been tested by the manufacturer and verified independently by previous published studies. Validation profiles and relevant citations can be found in the links provided.

Animals and other research organisms

Policy information about studies involving animals; ARRIVE guidelines recommended for reporting animal research, and Sex and Gender in Research

Laboratory animals	C57BL/6J inbred male mice (JAX no. 000664; CD45.l-CD45.2+) and B6.SJL-Ptprca Pepcb/BoyJ male mice (JAX no. 002014; CD45.l+ CD45.2-) were housed in the Harvard Biolabs Animal Facility under standard conditions. On the day of sacrifice, young mice were 3-4 months (13-15 weeks) of age, and old mice were 20-22 months (80-87 weeks) of age.
Wild animals	There were no wild animals.
Reporting on sex	All mice were male.
Field-collected samples	There were no field-collected samples.
Ethics oversight	ll experimental procedures were approved in advance by the Institutional Animal Care and Use Committee of Harvard University (AEP no. 10-23) and are in compliance with federal and state laws.

Note that full information on the approval of the study protocol must also be provided in the manuscript.

Flow Cytometry

Plots

Confirm that:

☒ The axis labels state the marker and fluorochrome used (e.g. CD4-FITC).

☒ The axis scales are clearly visible. Include numbers along axes only for bottom left plot of group (a 'group' is an analysis of identical markers).

☒ All plots are contour plots with outliers or pseudocolor plots.

☒ A numerical value for number of cells or percentage (with statistics) is provided.

Methodology

Sample preparation	For the flow cytometry experiments we used single cell suspensions of spleens derived from the young and old parabionts
Instrument	BD LSR II Flow Cytometer (BD Biosciences)
Software	Data were analyzed using the FlowJo software (version 10)

Cell population abundance	Total sorted cells per spleen: 120,000-150,000 CD45.1+ splenocytes: 20-35,000 cells CD45.2+ splenocytes: 20-35,000 cells
Gating strategy	Gates were set manually by using compensation beads and appropriate control samples

☒ Tick this box to confirm that a figure exemplifying the gating strategy is provided in the Supplementary Information.
