# Supplementary figures and images for: Heterochronic parabiosis reprograms the mouse brain transcriptome by shifting aging signatures in multiple cell types
Source: Nat Aging. 2023 Mar 9;3(3):327–45. doi: 10.1038/s43587-023-00373-6 (PMC10154248; doi:10.1038/s43587-023-00373-6)

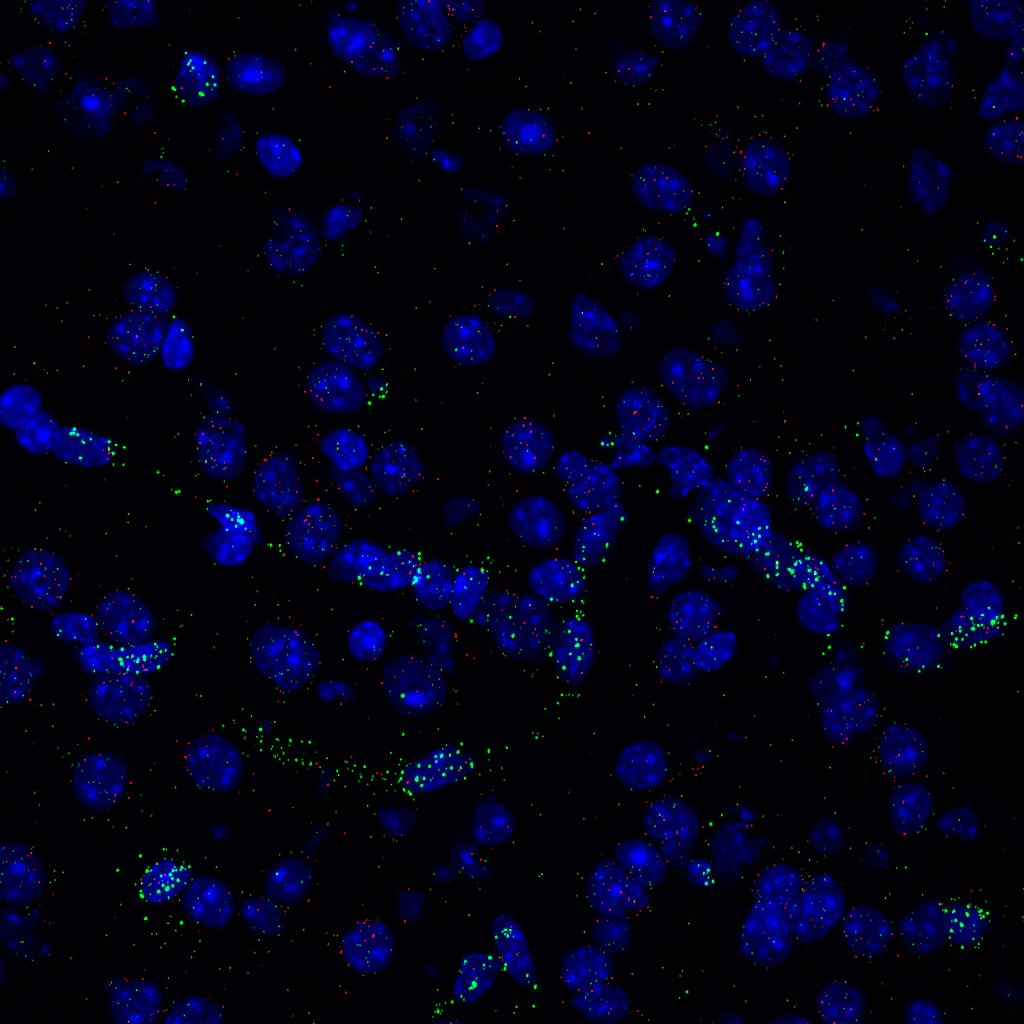

Supplement: Supplementary file 23 — Raw images of RNA in situ hybridization for Klf6 (a) YX. [file 43587_2023_373_MOESM23_ESM.tiff]

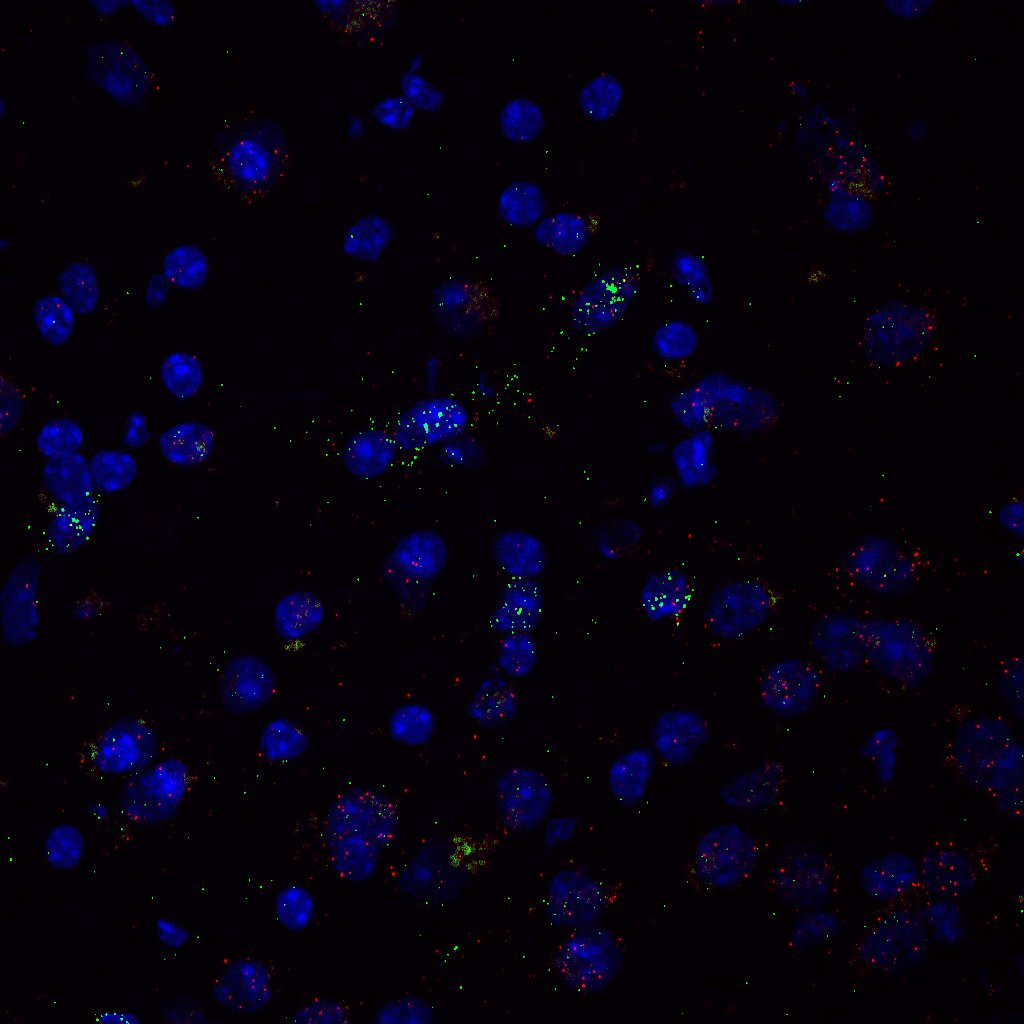

Supplement: Supplementary file 24 — Raw images of RNA in situ hybridization for Klf6 (a) OY. [file 43587_2023_373_MOESM24_ESM.tiff]

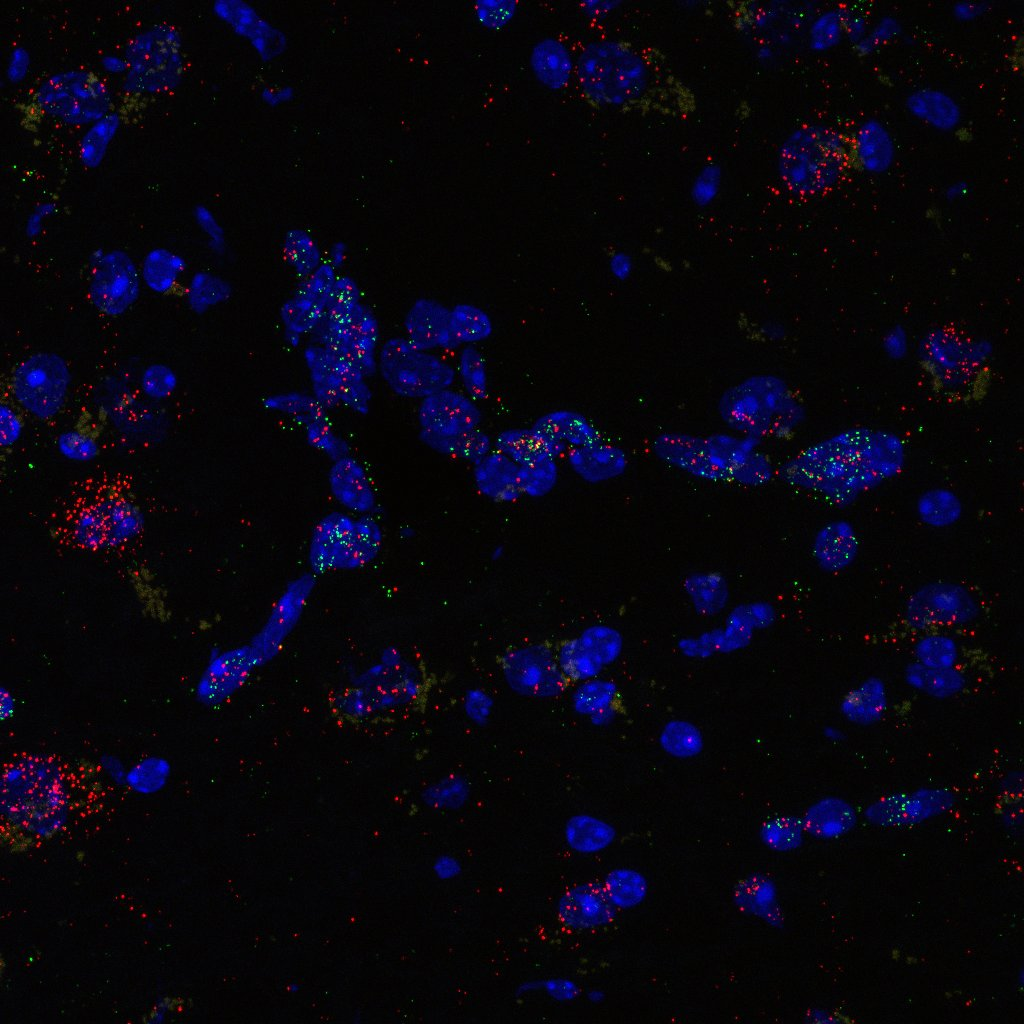

Supplement: Supplementary file 25 — Raw images of RNA in situ hybridization for Klf6 (a) OO. [file 43587_2023_373_MOESM25_ESM.tiff]

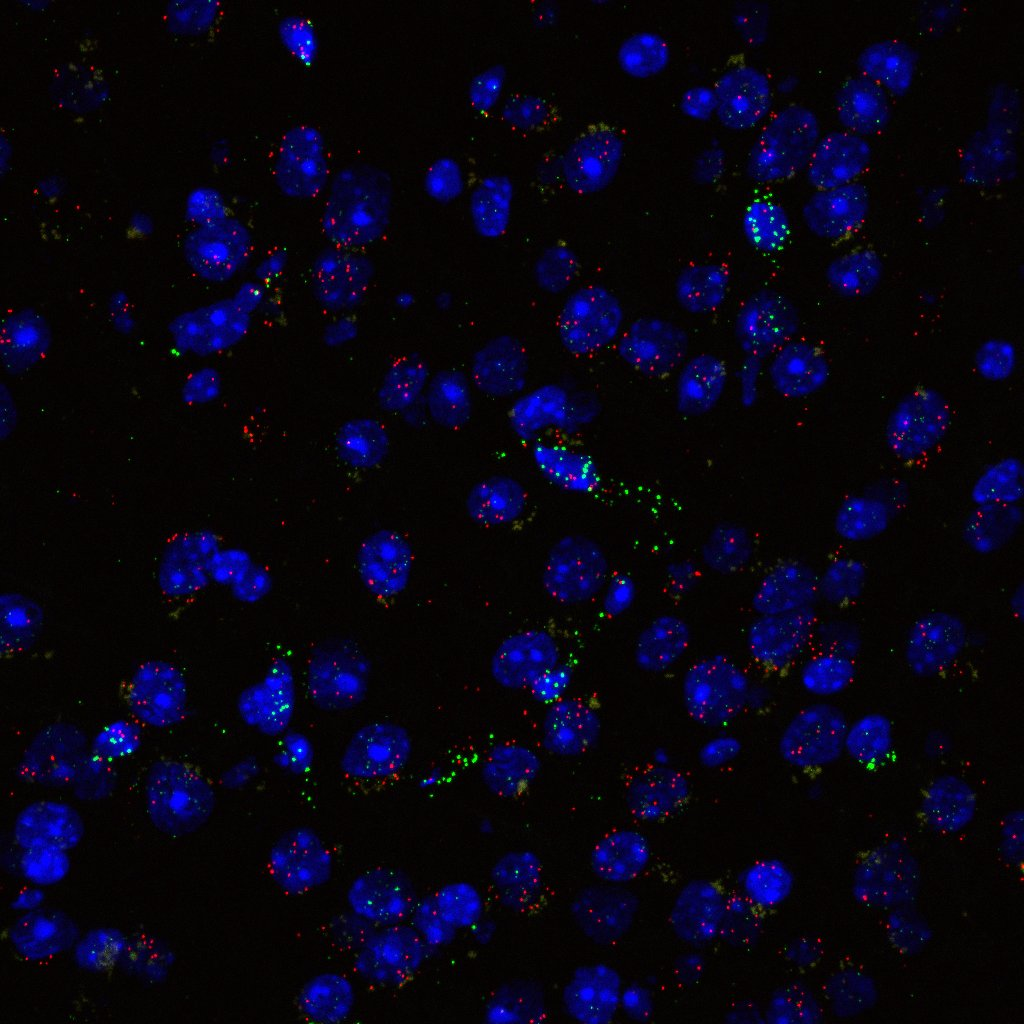

Supplement: Supplementary file 26 — Raw images of RNA in situ hybridization for Klf6 (a) OX. [file 43587_2023_373_MOESM26_ESM.tiff]

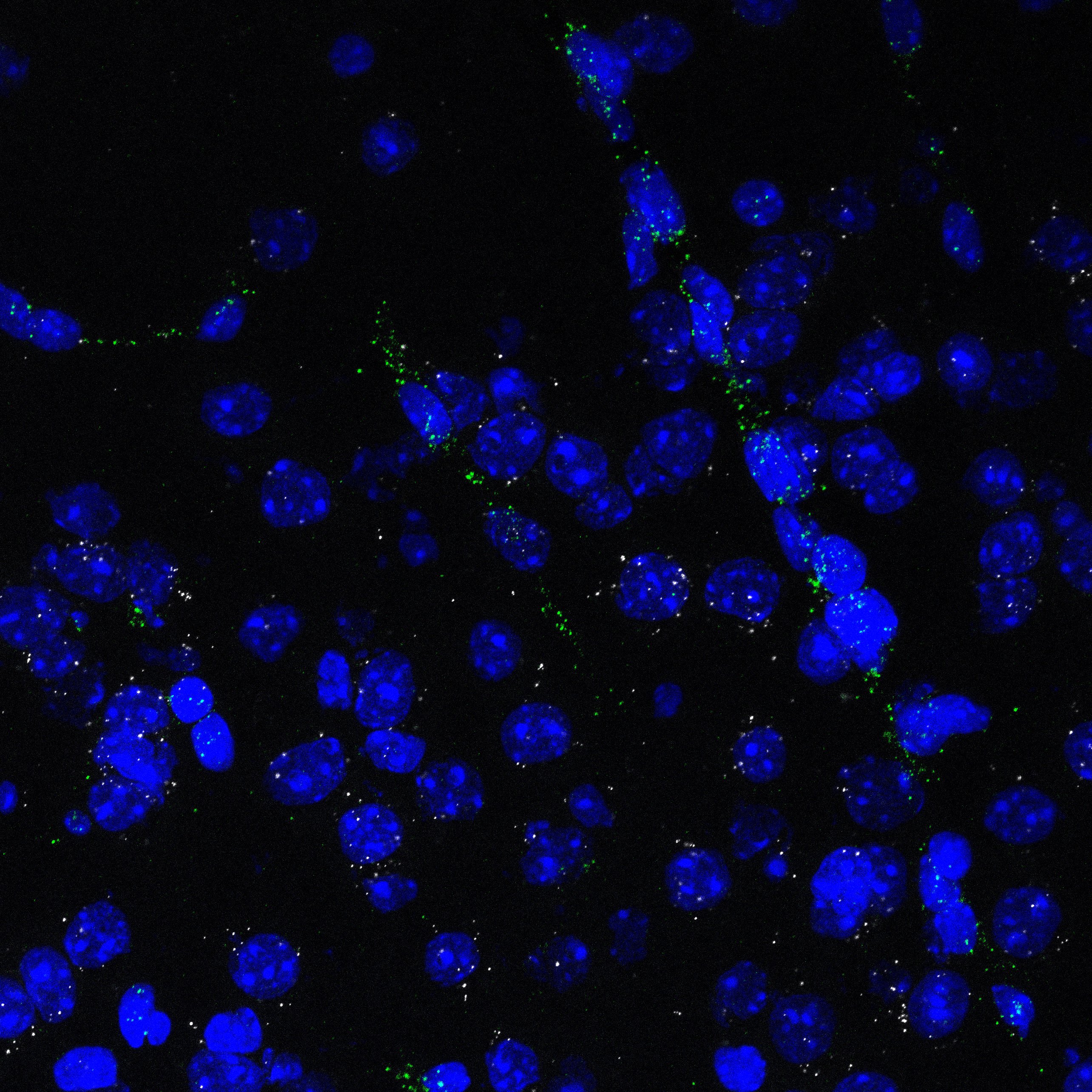

Supplement: Supplementary file 27 — Raw images of RNA in situ hybridization for Hspa1a (c) YX. [file 43587_2023_373_MOESM27_ESM.jpg]

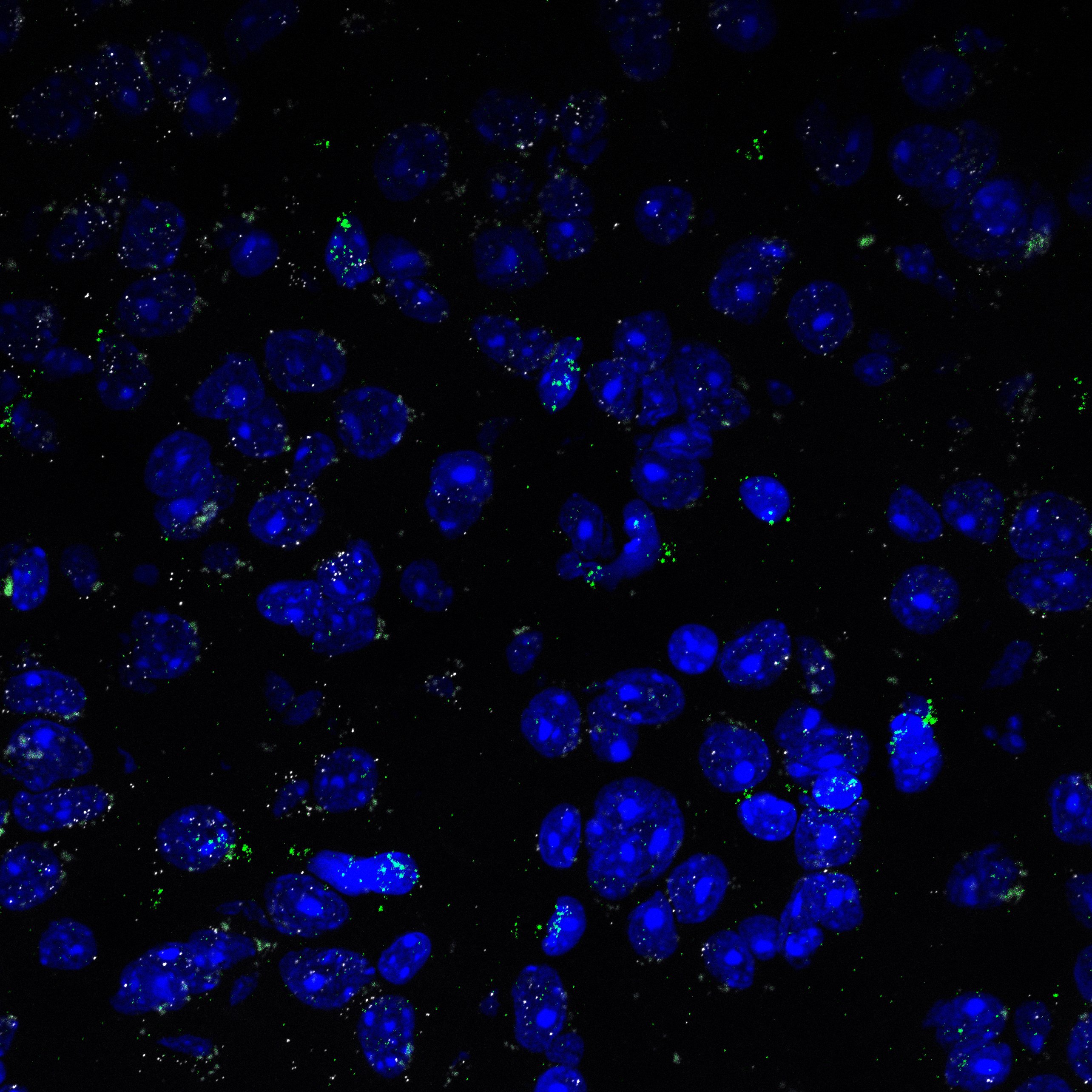

Supplement: Supplementary file 28 — Raw images of RNA in situ hybridization for Hspa1a (c) OY. [file 43587_2023_373_MOESM28_ESM.jpg]

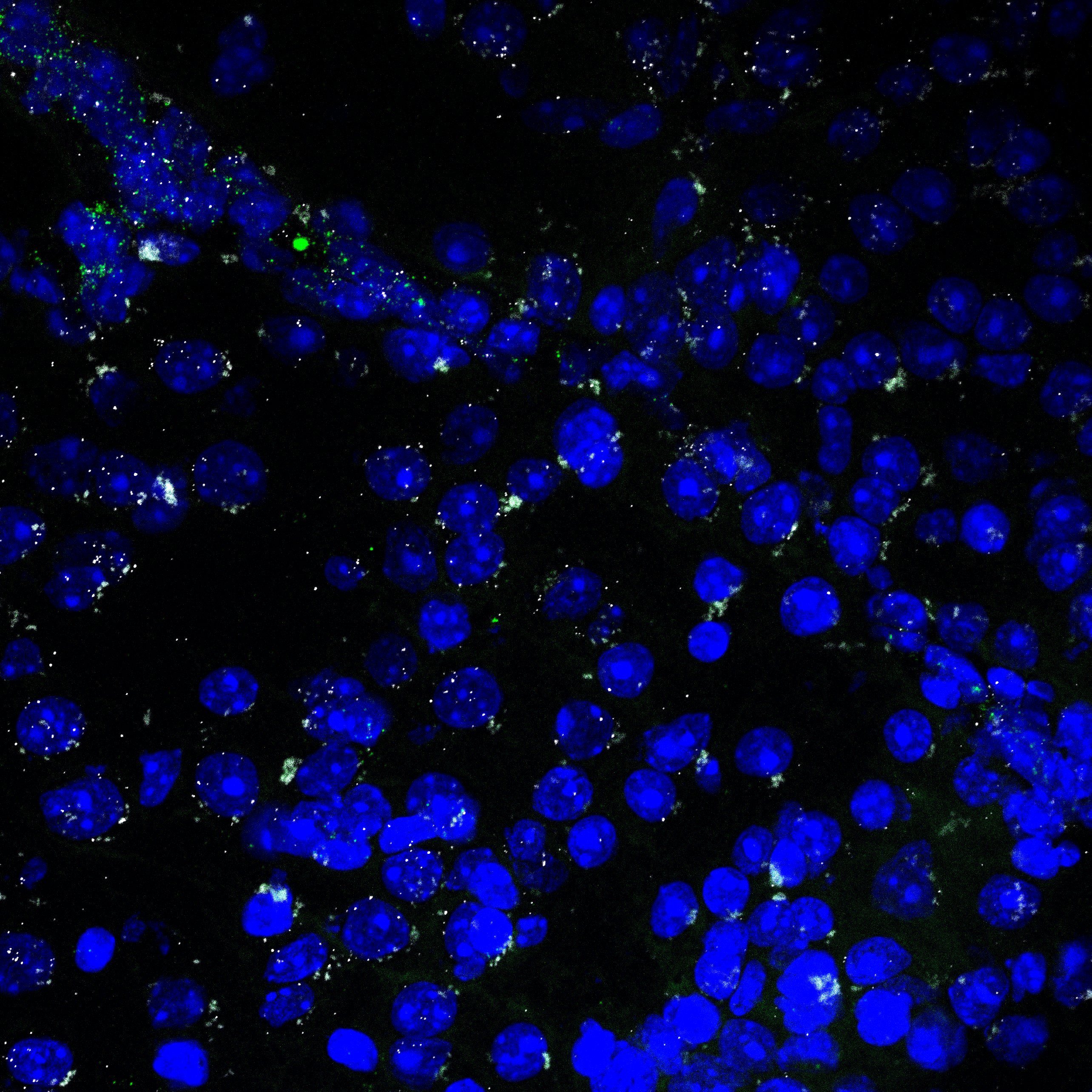

Supplement: Supplementary file 29 — Raw images of RNA in situ hybridization for Hspa1a (c) OO. [file 43587_2023_373_MOESM29_ESM.jpg]

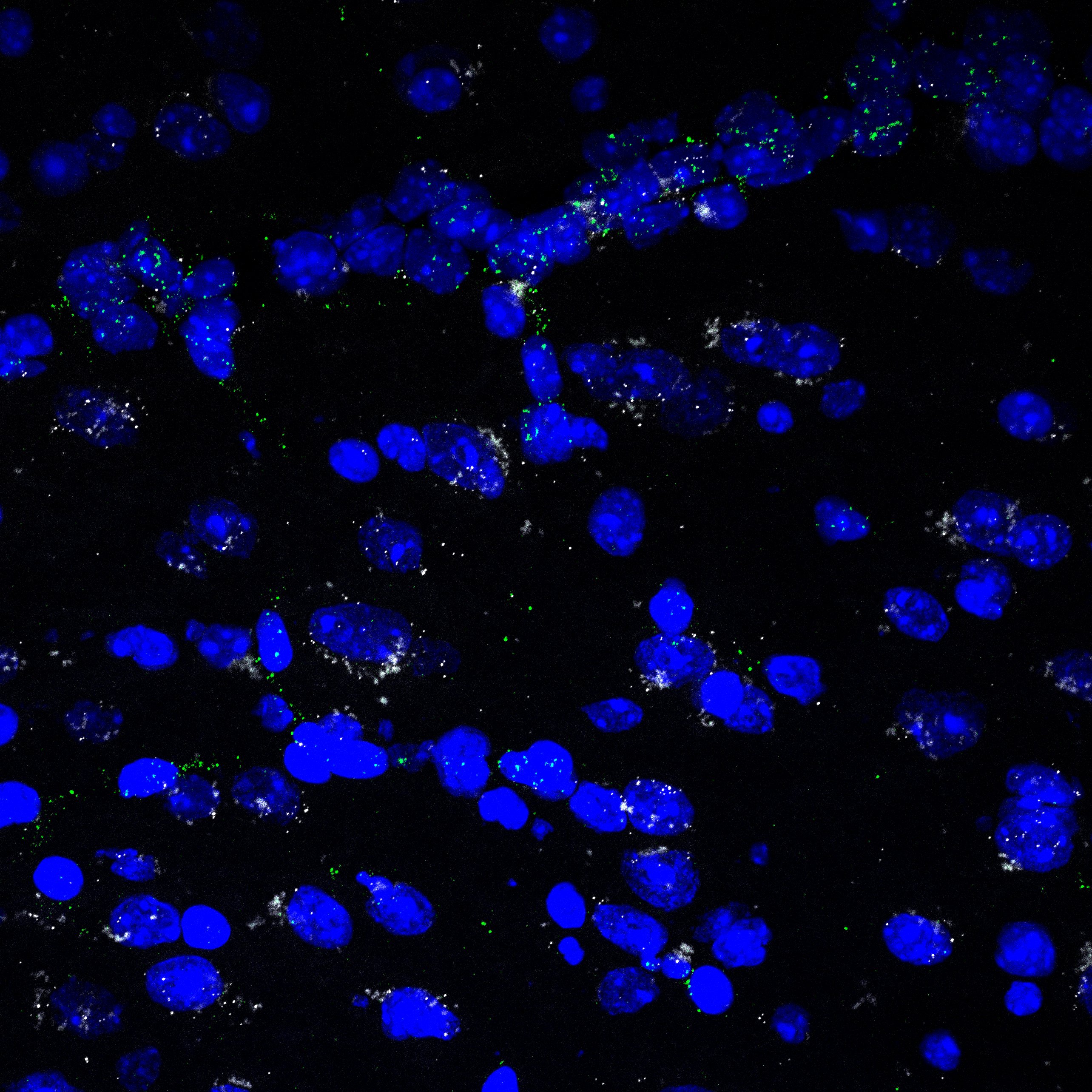

Supplement: Supplementary file 30 — Raw images of RNA in situ hybridization for Hspa1a (c) OX. [file 43587_2023_373_MOESM30_ESM.jpg]

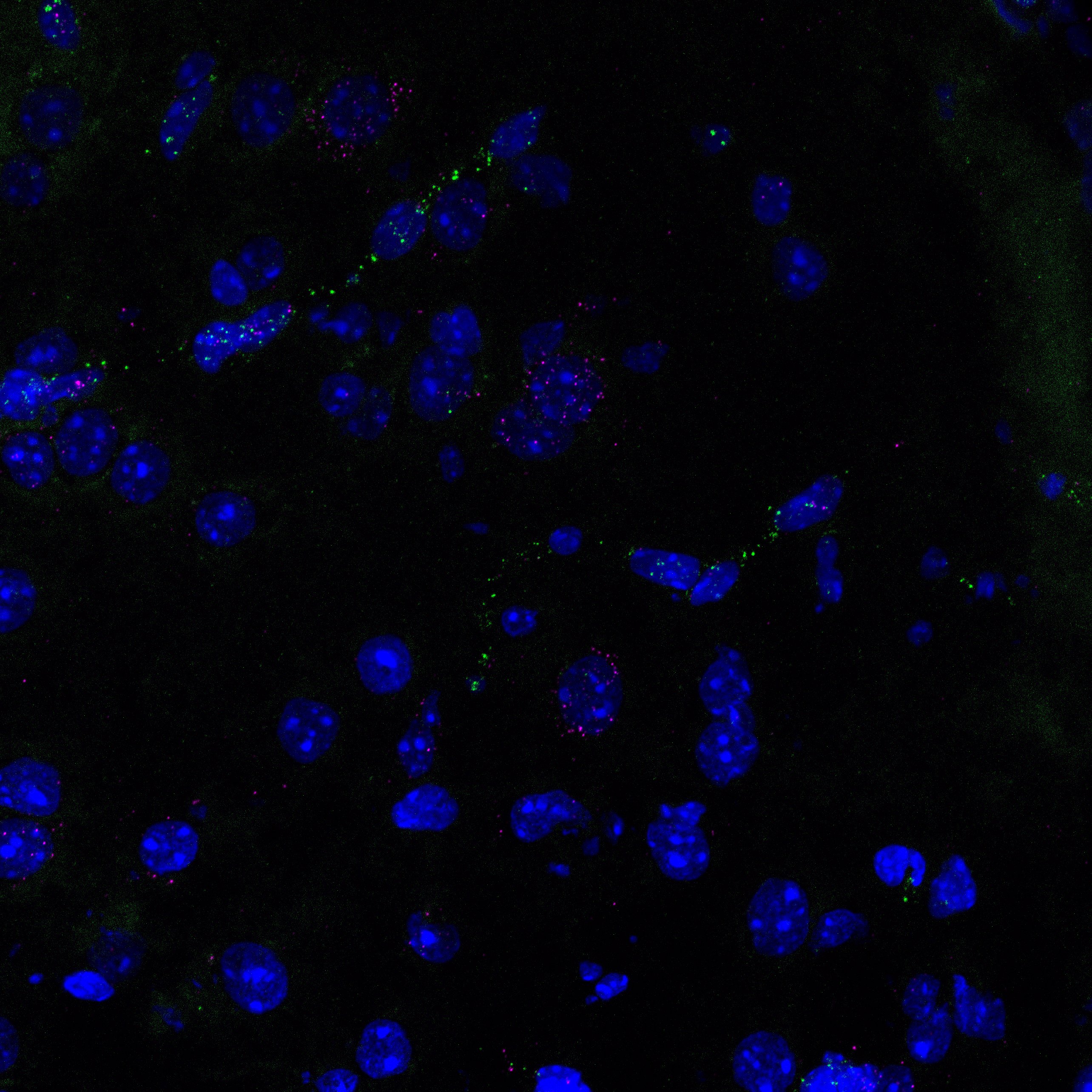

Supplement: Supplementary file 31 — Raw images of RNA in situ hybridization for Cdkn1a YX. [file 43587_2023_373_MOESM31_ESM.jpg]

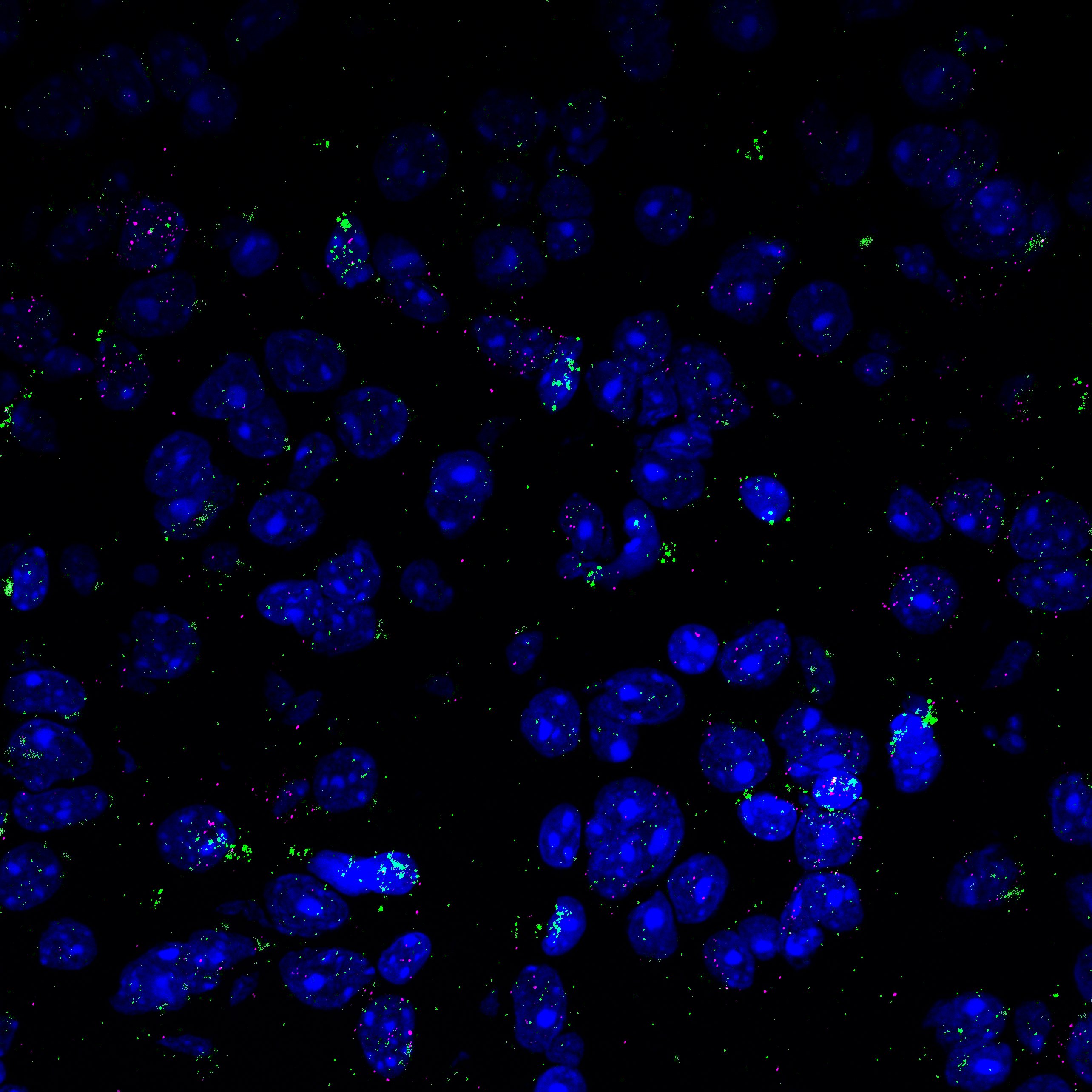

Supplement: Supplementary file 32 — Raw images of RNA in situ hybridization for Cdkn1a OY. [file 43587_2023_373_MOESM32_ESM.jpg]

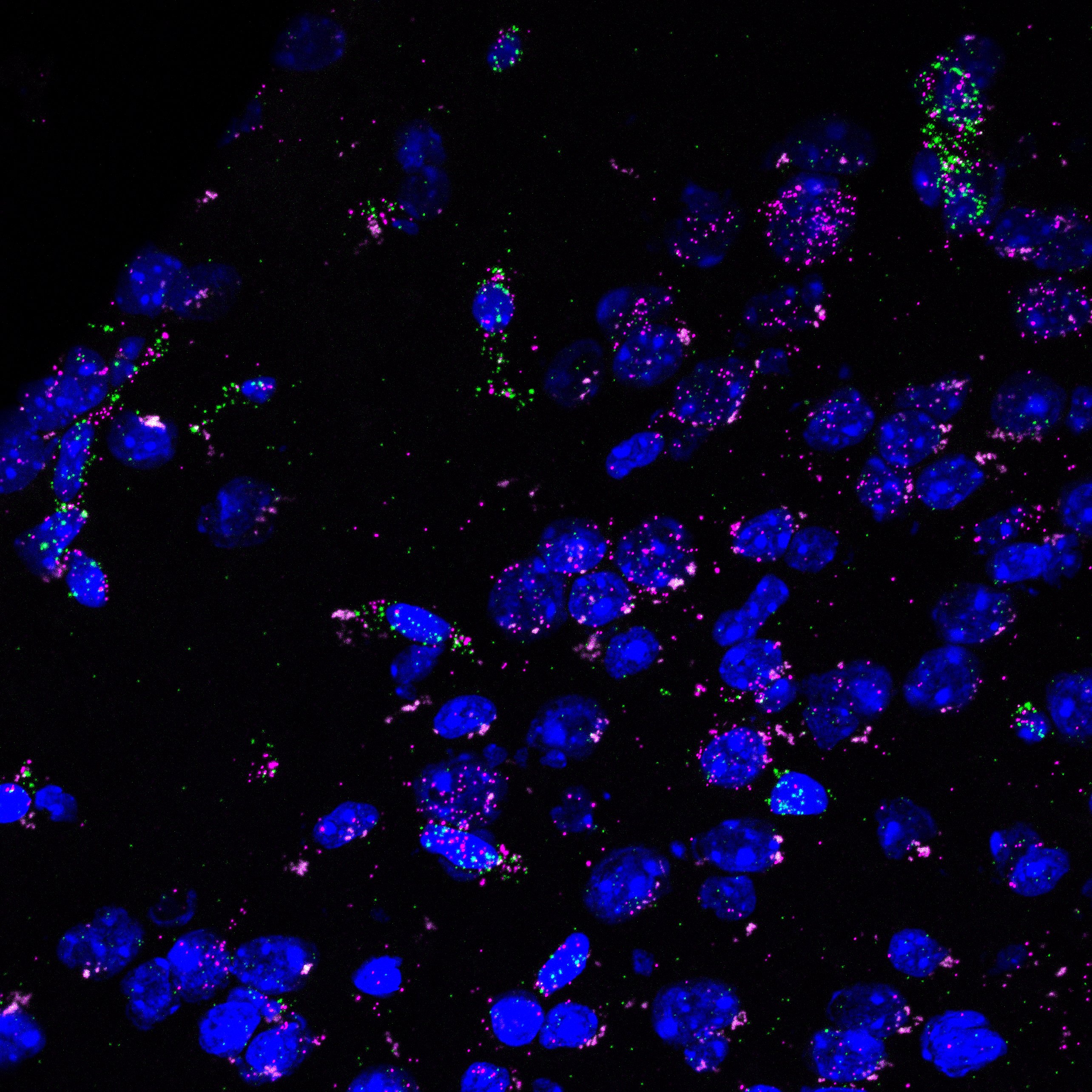

Supplement: Supplementary file 33 — Raw images of RNA in situ hybridization for Cdkn1a OO. [file 43587_2023_373_MOESM33_ESM.jpg]

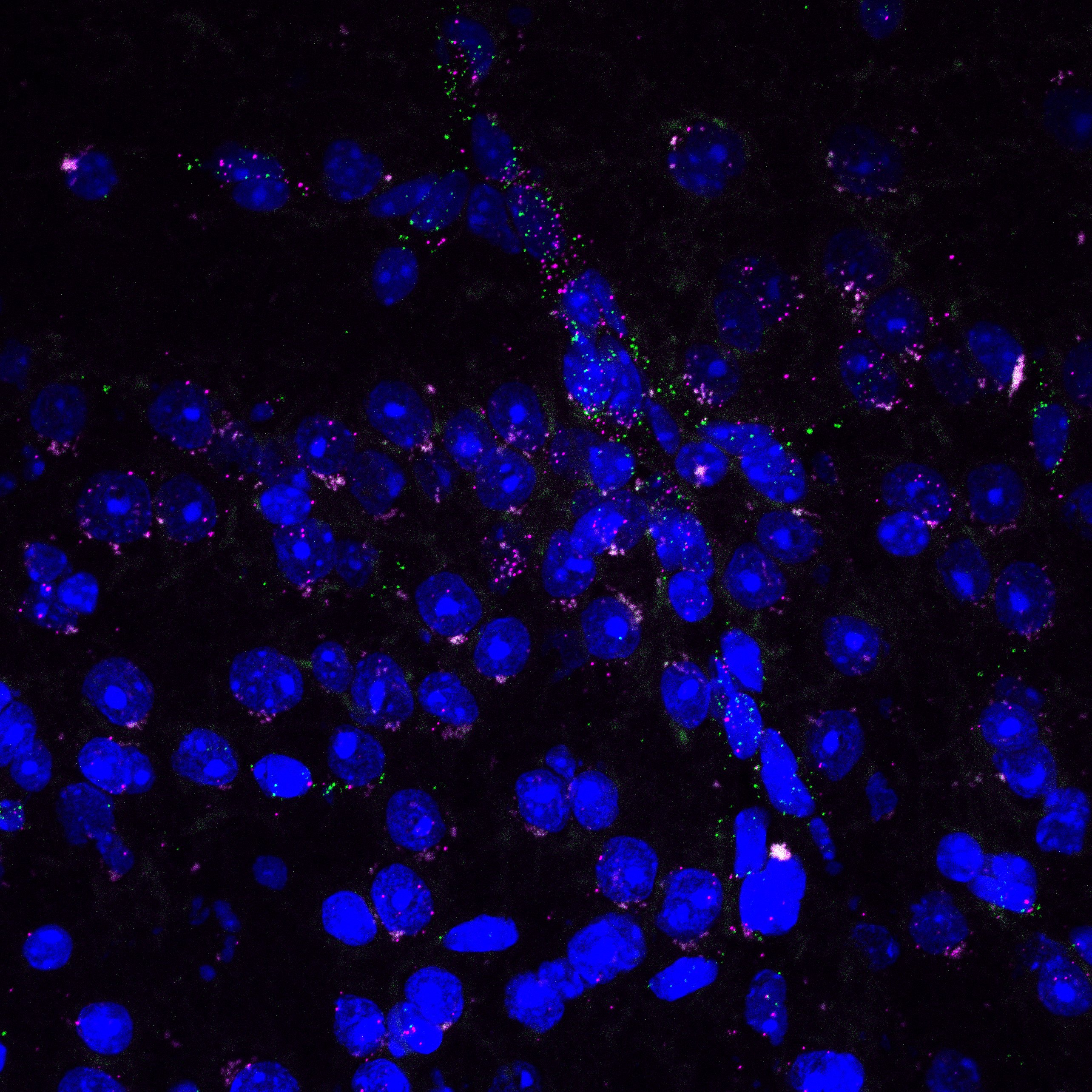

Supplement: Supplementary file 34 — Raw images of RNA in situ hybridization for Cdkn1a OX. [file 43587_2023_373_MOESM34_ESM.jpg]
