# Supplementary material for: Author Correction: Heterochronic parabiosis reprograms the mouse brain transcriptome by shifting aging signatures in multiple cell types
Source: Nat Aging. 2025 Jan 29;5(2):333. doi: 10.1038/s43587-025-00804-6 (PMC11839456; doi:10.1038/s43587-025-00804-6)

nature aging

Supplementary information

Author Correction: Heterochronic parabiosis reprograms the mouse brain transcriptome by shifting aging signatures in multiple cell types

In the format provided by the authors and unedited

Original image with 3 probes showing the individual channels.

OO: Uncropped image

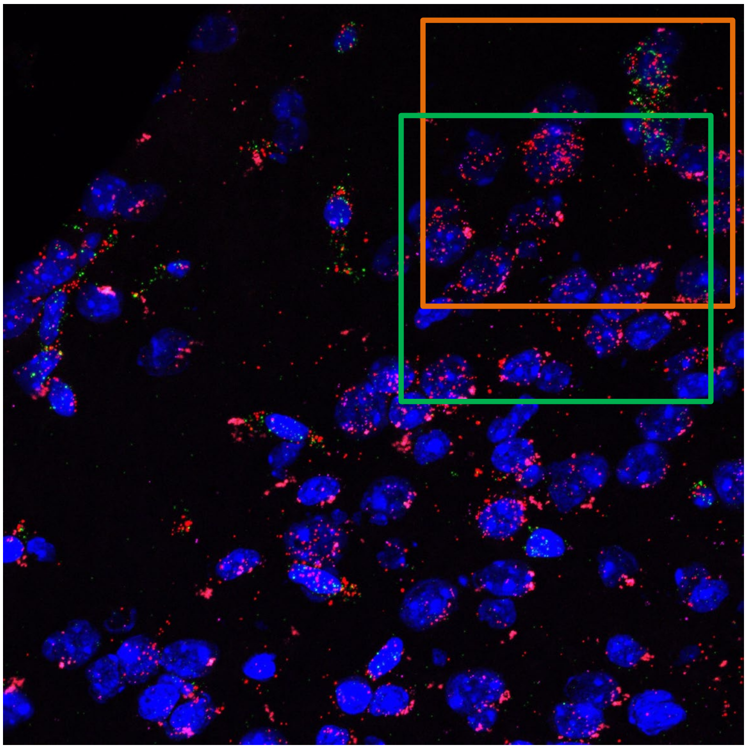

DAPI Hspa1a Pecam1 Cdkn1a

Individual channels

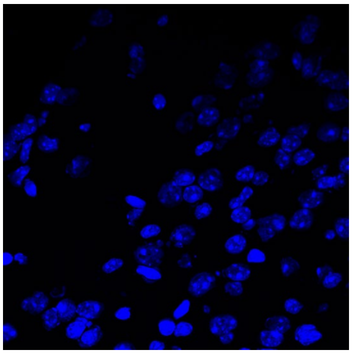

DAPI

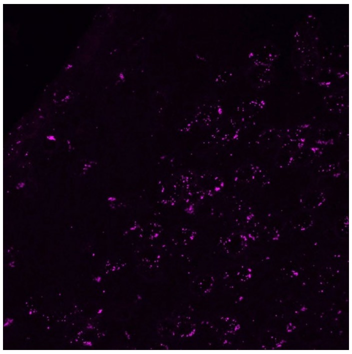

Hspa1a

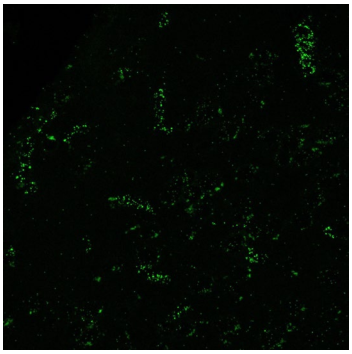

Pecam1

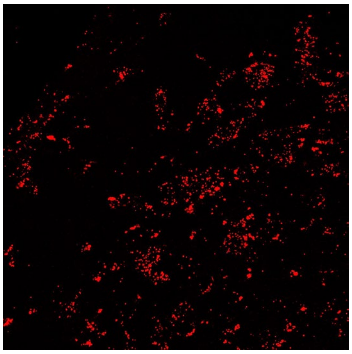

Cdkn1a

Images used in the manuscript, cropped from the original (top).

Fig.5c
OO: Cropped image in the manuscript

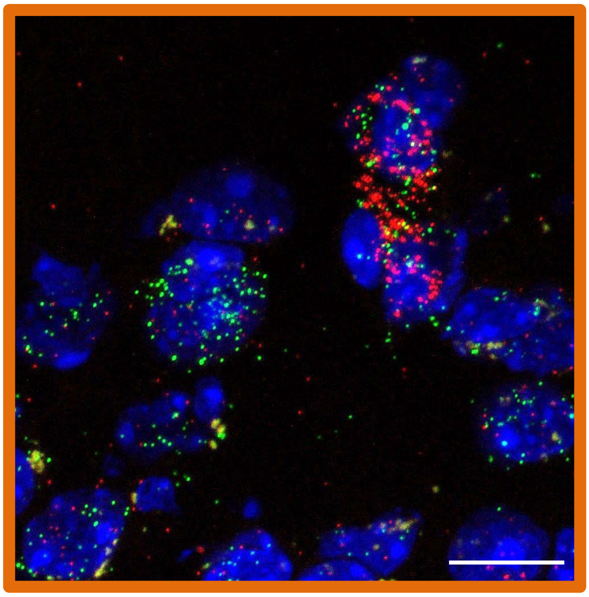

DAPI Pecam1 Hspa1a

Fig.8b
OO: Cropped image in the manuscript

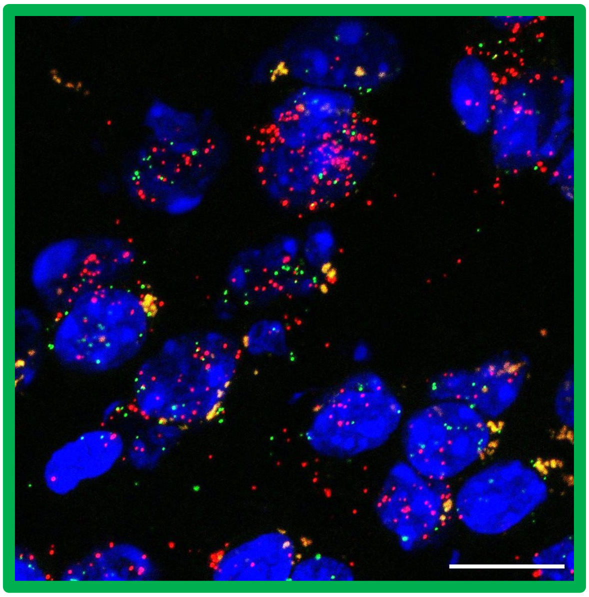

DAPI Pecam1 Cdkn1a

Comparison of new images (bottom) to original images in manuscript (top). Suspected duplication highlighted in the yellow boxes.

Fig.5c

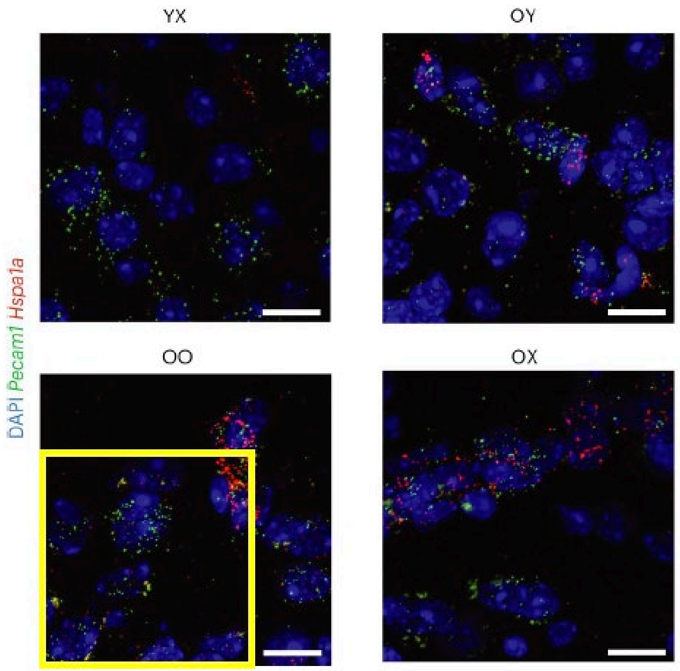

Updated version

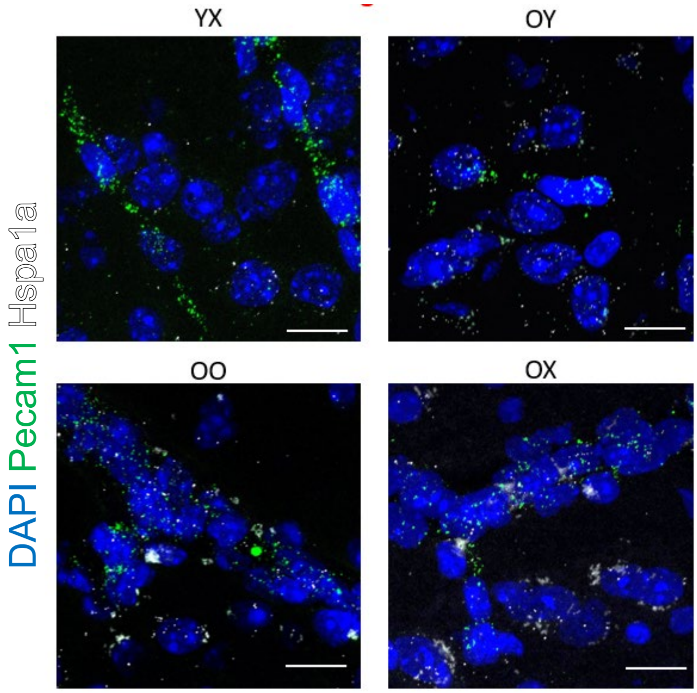

Fig.8b

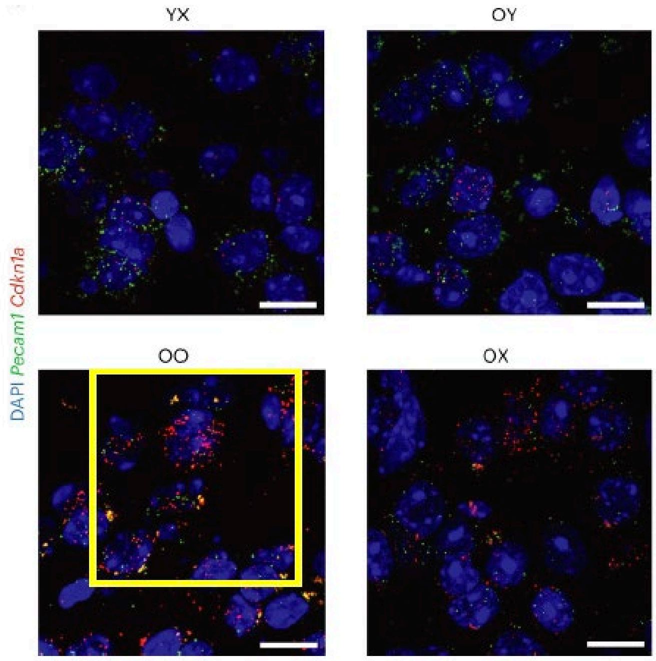

Updated version

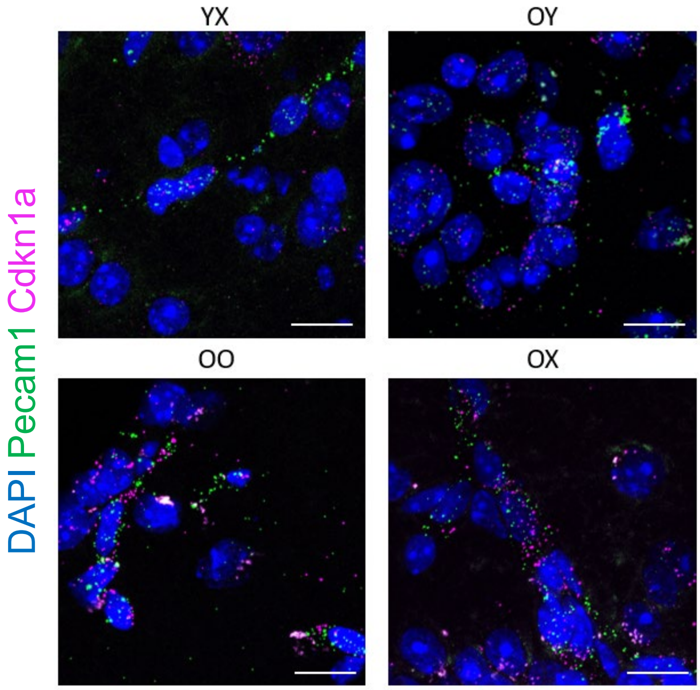

Supplement: Supplementary file 1 — Supplementary Data [file 43587_2025_804_MOESM1_ESM.pdf]
